# Association Between the Chinese Visceral Adiposity Index-Frailty Index (CVAI-FI) and the Incidence of New-Onset Stroke in Individuals with Cardiovascular-Kidney-Metabolic Syndrome Stage 0–3: Evidence From a National Population-Based Study

**DOI:** 10.5334/gh.1597

**Published:** 2026-10-22

**Authors:** Enfa Zhao, Hang Xie, Bingtian Dong, Chaoxue Zhang

**Affiliations:** 1 Department of Ultrasound, the First Affiliated Hospital of Anhui Medical University, Hefei 230022, Anhui Province, China; 2 Department of Cardiovascular Medicine, The First Affiliated Hospital of Xi’an Jiaotong University, Xi’an, Shaanxi, 710061, China

**Keywords:** Chinese visceral adiposity index, Frailty index, Cardiovascular-Kidney-Metabolic syndrome, Stroke, CHARLS, Cumulative exposure

## Abstract

**Objective:**

Cardiovascular-Kidney-Metabolic (CKM) syndrome represents a multisystem disorder linking metabolic dysfunction with cardiovascular complications. While the Chinese Visceral Adiposity Index (CVAI) and Frailty Index (FI) independently predict adverse outcomes, their combined utility for stroke prediction across CKM syndrome stages 0–3 remains unexplored.

**Methods:**

We analyzed 6,772 participants aged ≥45 years from the China Health and Retirement Longitudinal Study (2011–2020). Participants were classified into CKM stages 0–3, excluding stage 4 or baseline stroke. CVAI was calculated using age, BMI, waist circumference, triglycerides, and HDL-C. FI incorporated 32 health deficits following standardized methodology. The CVAI-FI index was derived as the product of CVAI and FI. Cumulative exposure was assessed in 4,170 participants with measurements from Waves 1 (2011) and 3 (2015). Multivariable logistic regression evaluated associations with incident stroke, adjusting for sociodemographic, behavioral, and clinical confounders. Model performance was assessed using ROC curves, with incremental value determined through NRI and IDI. Restricted cubic splines characterized dose-response relationships.

**Results:**

During follow-up, 447 participants (6.6%) developed stroke. CVAI-FI demonstrated a significant dose-response association with stroke risk (*P* for nonlinearity <0.001). Each one-SD increment in CVAI-FI corresponded to 31% higher stroke risk (OR 1.31, 95% CI 1.20–1.43). Cumulative CVAI-FI achieved superior discrimination (AUC 0.675) compared to standalone CVAI (0.616), FI (0.609), and baseline CVAI-FI (0.641, all P < 0.001), with significant IDI (0.018–0.020) and NRI (0.032) improvements. Associations remained robust across subgroups, with stronger effects among individuals with secondary education or above (OR 1.83) and those without hypertension (OR 1.51).

**Conclusions:**

The CVAI-FI index provides enhanced stroke risk stratification across CKM syndrome stages 0–3, with quantifiable improvements in discrimination and reclassification. Cumulative exposure assessment further strengthens predictive accuracy. These findings support CVAI-FI’s clinical utility for identifying high-risk individuals requiring intensified preventive interventions within emerging CKM syndrome frameworks.

## Graphic Abstract

**Figure:**
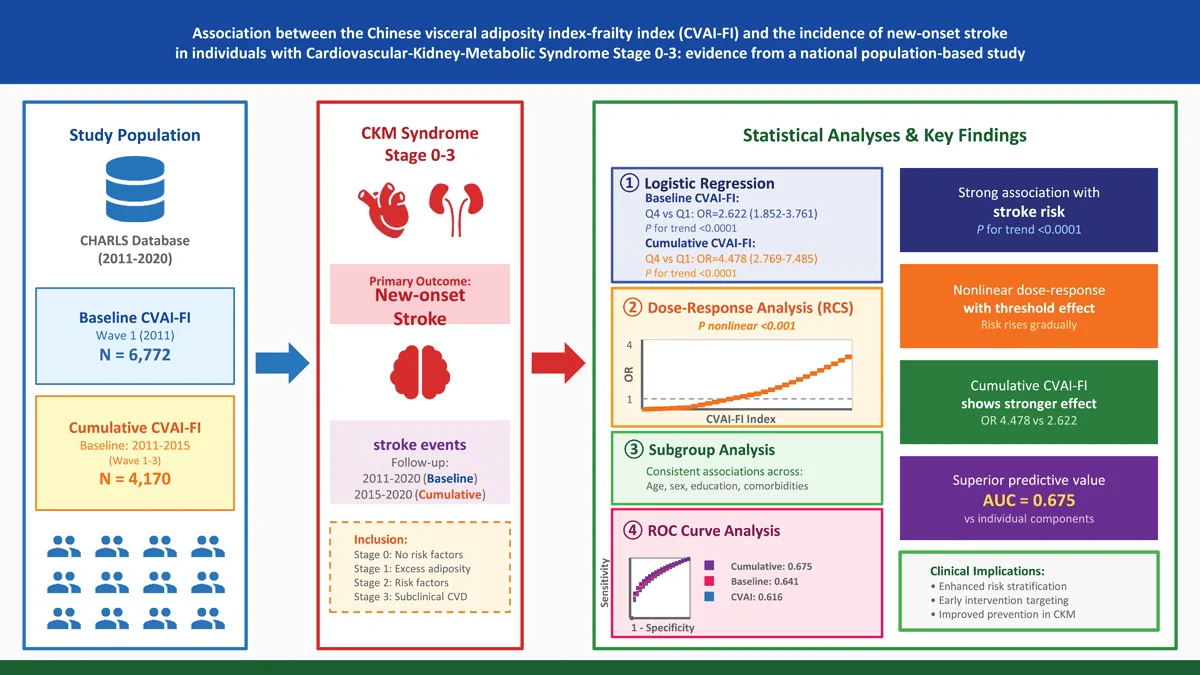


## Introduction

As the international burden of cardiovascular disorders intensifies, stroke maintains its position as a major driver of death and disability across diverse populations (1). In China, the burden of stroke is particularly substantial, with stroke serving as a major contributor to cardiovascular disease (CVD)-related mortality and disability (2). Emerging evidence increasingly recognizes that metabolic disorders, CVD, and chronic kidney disease (CKD) are not isolated clinical entities but rather closely interconnected conditions sharing common pathophysiological mechanisms (3,4). In October 2023, the AHA formalized this conceptual shift through introduction of the CKM syndrome classification system (5). This syndrome is characterized as a multiorgan disorder generated by pathophysiological convergence of metabolic risk states (including excess adiposity and glycemic dysregulation), chronic renal disease, and cardiovascular pathologies (encompassing cardiac decompensation, stroke, coronary disease, and peripheral vascular pathology). The framework stratifies disease progression into five distinct stages (0–4) (5). Population-based data reveal that approximately 90% of American adults during the 2011–2020 period fulfilled classification criteria for CKM syndrome at stage 1 or beyond (6), highlighting its substantial public health impact. Importantly, CKM syndrome progression follows a modifiable trajectory, with appropriately directed prevention strategies capable of inducing remission or backward transition to less advanced stages (7,8,9), thus highlighting the essential role of timely risk profiling and early therapeutic action among populations within CKM syndrome stages 0 through 3.

Visceral adiposity and frailty represent two increasingly recognized risk factors with significant implications for cardiovascular health. Beyond serving merely as an energy reservoir, visceral adipose tissue operates as a metabolically active endocrine entity that releases diverse bioactive molecules and pro-inflammatory cytokines, thereby driving insulin resistance, vascular endothelial impairment, and atherogenic progression (10,11). The Chinese visceral adiposity index (CVAI), specifically developed and validated for the Chinese population, provides a practical assessment tool that incorporates age, body mass index, waist circumference, triglycerides, and high-density lipoprotein cholesterol to estimate visceral fat accumulation (12,13,14,15). Concurrently, frailty—characterized by decreased physiological reserve and increased vulnerability to stressors—has emerged as an independent predictor of adverse cardiovascular outcomes, including stroke (16,17). The frailty index (FI), which quantifies the accumulation of health deficits across multiple domains, has demonstrated robust prognostic value for cardiovascular events (18,19). While both visceral adiposity and frailty independently predict cardiovascular risk, their potential synergistic effects on stroke incidence have not been systematically investigated. Given that metabolic dysregulation constitutes a fundamental pathophysiological mechanism linking CKM syndrome components (5), and considering that both visceral adiposity and frailty reflect distinct yet interconnected aspects of metabolic and physiological vulnerability, examining their combined association with new-onset stroke risk in the CKM individual represents a clinically meaningful research priority.

To bridge this knowledge deficit, we analyzed prospective CHARLS data to evaluate whether the composite CVAI-FI index predicts stroke development among individuals within early-to-intermediate CKM syndrome (stages 0–3). We hypothesized that increased CVAI-FI levels, reflecting the compounded burden of visceral adiposity and frailty, would show independent association with elevated stroke risk in this population. Additionally, we explored whether cumulative exposure to elevated CVAI-FI over time would demonstrate stronger predictive value than single-point assessment. Through comprehensive analyses including dose-response modeling using restricted cubic splines, extensive subgroup stratification, and predictive performance evaluation via receiver operating characteristic curves, we sought to provide robust evidence-based insights for early identification of high-risk participants and inform targeted prevention programs within the CKM syndrome framework.

## Methods

### Study design and population

Our analysis leveraged the CHARLS database, a nationwide longitudinal survey designed to systematically capture detailed health, financial, and sociocultural data from middle-aged and older Chinese adults (≥45 years). The CHARLS employed a multistage, stratified, probability-proportional-to-size sampling methodology, covering 450 villages and urban communities across 28 provinces in mainland China (20). The baseline survey was conducted in 2011–2012 (Wave 1, n = 17,705), with subsequent follow-up waves completed in 2013 (Wave 2), 2015 (Wave 3), 2018 (Wave 4), and 2020 (Wave 5). All study procedures were conducted in accordance with the Declaration of Helsinki and received ethical approval from the Biomedical Ethics Review Committee of Peking University (IRB00001052-11015). Participants provided written informed consent subsequent to thorough explanation of study purposes and operational procedures. Detailed accounts of CHARLS survey design, sampling methodology, and data acquisition procedures are documented in earlier publications (20).

For the present analysis, two distinct analytical cohorts were constructed to examine both baseline and cumulative associations between CVAI-FI and stroke incidence (Figure 1). The baseline cohort utilized Wave 1 (2011) as the exposure assessment timepoint and Waves 2–5 (2013–2020) as the outcome ascertainment period. From the initial 17,705 participants enrolled in Wave 1, we implemented the following stepwise exclusion procedures: 1) participants younger than 45 years or with absent age information (n = 393); 2) missing sex information (n = 12); 3) incomplete data required to calculate body mass index (BMI), waist circumference (WC), triglycerides (TG), or high-density lipoprotein cholesterol (HDL-C) (n = 7,947); 4) missing frailty index data (n = 7); 5) absent CKM syndrome classification data or diagnosis of stage 4 CKM disease (n = 2,375); and 6) prevalent stroke at Wave 1 or lost to follow-up (n = 199). Following implementation of these exclusion procedures, 6,772 individuals met eligibility requirements for the baseline CVAI-FI analytical cohort.

**Figure 1:**
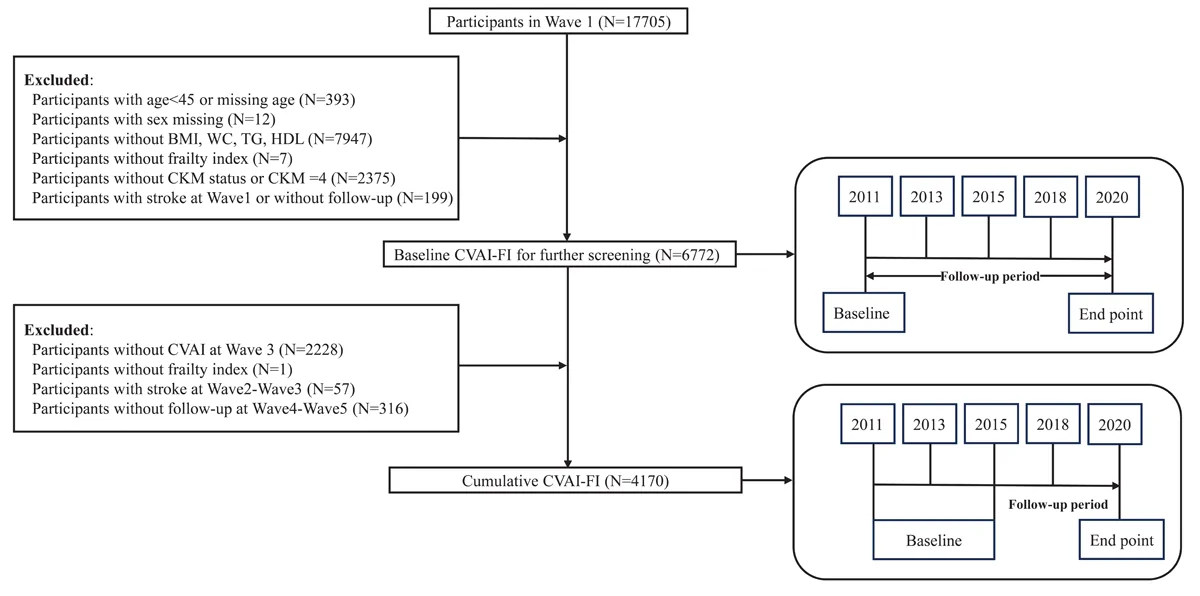
Participant selection flowchart. From 17,705 initial CHARLS participants, 6,772 were eligible for baseline CVAI-FI analysis (2011–2020) and 4,170 for cumulative analysis (2015–2020) after applying exclusion criteria shown in the flowchart. Abbreviations: CVAI-FI, Chinese visceral adiposity index-frailty index; CKM, cardiovascular-kidney-metabolic; BMI, body mass index; WC, waist circumference; TG, triglycerides; HDL, high-density lipoprotein cholesterol.

To assess the impact of sustained exposure to elevated CVAI-FI levels, we constructed a cumulative exposure cohort using CVAI-FI measurements from both Wave 1 (2011) and Wave 3 (2015), with outcome ascertainment during Waves 4–5 (2018–2020). Starting from the 6,772 participants in the baseline cohort, we further excluded individuals with: 1) missing CVAI data at Wave 3 (n = 2,228); 2) missing frailty index data at Wave 3 (n = 1); 3) incident stroke occurring between Wave 2 and Wave 3 (n = 57); and 4) loss to follow-up before Wave 4 or Wave 5 (n = 316). This yielded a final analytical sample of 4,170 participants for the cumulative CVAI-FI analysis. The cumulative CVAI-FI was calculated as the average of CVAI-FI values measured at Wave 1 (2011) and Wave 3 (2015), providing an integrated measure of sustained cardiometabolic and frailty burden over the early follow-up period. The participant selection process for both analytical cohorts is illustrated in detail in Figure 1.

### Assessment of CVAI-FI and cumulative CVAI-FI

CVAI was derived through application of sex-stratified equations previously verified for use in Chinese cohorts (21). The formulas are as follows:


$$\begin{array}{c} \text{For men: CVAI}=-267.93+0.68\times \text{age}\left(\text{years}\right)+0.03\times \text{BMI}\left(\text{kg}/\text{m}²\right)+4.00\times \text{WC}\left(\text{cm}\right)\\ \;+22.00\times {\text{log}}_{10}\left(\text{TG}\left(\text{mmol}/\text{L}\right)\right)-16.32\times \text{HDL-C}\left(\text{mmol}/\text{L}\right). \end{array}$$

$$\begin{array}{c} \text{For women: CVAI}=187.32+1.71\times \text{age}\left(\text{years}\right)+4.23\times \text{BMI}\left(\text{kg}/\text{m}²\right)+1.12\times \text{WC}\left(\text{cm}\right)\\ \;+39.76\times \log_{10}\left(\text{TG}\left(\text{mmol}/\text{L}\right)\right)-11.66\times \text{HDL-C}\left(\text{mmol}/\text{L}\right). \end{array}$$

The equations incorporate the following parameters: BMI (body mass index), WC (waist circumference), TG (triglycerides), and HDL-C (high-density lipoprotein cholesterol). All anthropometric measurements and biochemical parameters were obtained following standardized protocols as described in the CHARLS study documentation (20).

The FI was constructed using a deficit accumulation approach based on 32 health-related variables derived from the CHARLS database, as previously described (22,23). These variables encompassed five major domains: 1) chronic disease conditions (10 items: physician-diagnosed hypertension, diabetes, heart disease, arthritis, stroke, psychiatric problems, cancer, chronic lung disease, asthma, and memory-related disease); 2) functional limitations (13 items: activities of daily living including dressing, bathing, eating, bed mobility, and toilet use; instrumental activities of daily living including money management, medication taking, grocery shopping, meal preparation, and housework; mobility limitations including walking, chair rising, stair climbing, weight lifting, coin picking, stooping, and arm reaching); 3) self-assessed health status (1 item: general health rating); 4) sensory impairments (2 items: vision and hearing problems); 5) psychological and cognitive function (2 items: depressive symptoms assessed by the 10-item Center for Epidemiologic Studies Depression Scale (CESD-10) and cognitive performance measured by combined memory and orientation test scores). Each deficit variable was recoded into a binary format (0 = deficit absent, 1 = deficit present), except for cognitive function, which received continuous scoring from 0 to 1 calculated as: (memory test score + orientation test score)/14. The CESD-10 depression item was coded as 1 if the score exceeded 10, and 0 otherwise. Each individual’s FI was computed as the proportion of present deficits relative to the total number of assessed variables, producing a continuous index ranging from 0 to 1, where greater values indicate more pronounced frailty. In accordance with prior research, an FI threshold of ≥0.25 was used to classify frailty (24,25,26). Detailed descriptions of all 32 FI components and their scoring criteria are provided in Supplementary Table S1.

The CVAI-FI composite index was derived as the product of CVAI and FI (CVAI-FI = CVAI × FI) to reflect the combined burden of visceral adiposity and frailty on cardiovascular risk. This approach follows the precedent of other established multiplicative indices in cardiovascular epidemiology – such as the triglyceride-glucose–body mass index (TyG-BMI), TyG–waist circumference (TyG-WC), and TyG-CVAI – where the product of two risk components creates an integrated risk marker capturing complementary information from distinct physiological domains (27,28,29). To formally evaluate whether CVAI and FI interact multiplicatively beyond their independent main effects, we conducted a supplementary interaction analysis including CVAI, FI, and the CVAI × FI product term as separate terms in the fully adjusted regression model (Supplementary Table S2). Furthermore, to address the concern that the scale difference between CVAI (unbounded, range approximately –20 to 330) and FI (bounded 0–1) may cause CVAI to dominate the product, we conducted a sensitivity analysis comparing three alternative formulations: the original raw-scale product (CVAI × FI), a standardized product [z(CVAI) × z(FI)], and a sum-based composite [z(CVAI) + z(FI)], with results detailed in Supplementary Table S3. The original CVAI-FI and the sum-based composite demonstrated high consistency (r = 0.91) and comparable predictive performance, confirming that the index captures joint information from both components rather than merely reflecting scale dominance of CVAI alone. The CVAI-FI index was calculated at Wave 1 (2011) for all participants in the baseline cohort. The cumulative CVAI-FI was calculated as the area under the exposure-time curve using the trapezoidal rule, representing the cumulative burden of cardiometabolic-frailty exposure over the 4-year interval:


$$\text{Cumulative CVAI-FI}=\left[\left({\text{CVAI-FI}}_{2011}+{\text{CVAI-FI}}_{2015}\right)/2\right]\times \left(2015-2011\right)$$

This cumulative exposure metric, expressed in index-year units, captures the total burden of sustained cardiometabolic-frailty exposure over the 4-year period, accounting for potential temporal changes in both visceral adiposity and frailty status (30). The cumulative CVAI-FI metric captures prolonged exposure to elevated cardiometabolic-frailty burden, which may exert cumulative detrimental effects on cerebrovascular health beyond single-point assessment. The multiplicative formulation was selected as an integrated measure of visceral adiposity and frailty, consistent with published cardiovascular composite indices (27,28,29,31,32). These factors may provide complementary information through distinct pathways; however, the product formulation should not be interpreted as evidence of statistical or biological interaction.

### Definition of CKM syndrome stages 0 to 4

The CKM syndrome stages were defined according to the AHA Presidential Advisory framework integrating metabolic, cardiovascular, and renal parameters (5). For the present analysis, participants were operationally classified as follows: Stage 0: absence of overweight/obesity (BMI <23 kg/m², WC <90 cm (men)/<80 cm (women)), normal glucose metabolism (fasting glucose <100 mg/dL, HbA1c <5.7%), normal blood pressure (SBP <130 mmHg, DBP <80 mmHg, no antihypertensive medication), normal lipid profile (TG <150 mg/dL, HDL-C ≥40 mg/dL (men)/≥50 mg/dL (women)), and eGFR ≥60 mL/min/1.73 m², without CVD. Stage 1: excess adiposity (BMI ≥23 kg/m² or WC ≥90 cm (men)/≥80 cm (women)) or prediabetes (fasting glucose 100–125 mg/dL or HbA1c 5.7%–6.4%), without other metabolic risk factors or CVD. Stage 2: presence of metabolic risk factors (hypertriglyceridemia (TG ≥135 mg/dL), hypertension (SBP ≥130 mmHg or DBP ≥80 mmHg or antihypertensive use), diabetes (fasting glucose ≥125 mg/dL or HbA1c ≥6.5% or antidiabetic medication), or metabolic syndrome) and/or moderate CKD (eGFR 30–60 mL/min/1.73 m²), without CVD. Stage 3: subclinical CVD (elevated predicted 10-year CVD risk) or very high-risk CKD (eGFR <30 mL/min/1.73 m²), without clinical CVD. Stage 4: clinical CVD (self-reported heart disease or stroke); excluded from this analysis. Detailed cut-off values and definitions are provided in Supplementary Tables S4–S5.

### New-onset strokes and follow-up

The principal endpoint was incident stroke occurring during longitudinal follow-up. Stroke occurrence was identified via participant-reported physician diagnoses obtained through structured questionnaires at each survey wave. At baseline (Wave 1, 2011) and subsequent follow-up waves (Waves 2–5, spanning 2013–2020), participants were asked: ‘Have you been diagnosed with a stroke by a doctor?’ Those who responded affirmatively at follow-up but had not reported stroke at baseline were classified as having experienced new-onset stroke (33). For the baseline CVAI-FI analysis, individuals were followed from Wave 1 (2011) until the onset of stroke or the most recent follow-up wave (Wave 5, 2020), whichever came first. To ensure evaluation of only new-onset events, individuals with baseline stroke history were excluded from the baseline cohort. For the cumulative CVAI-FI analysis, a separate cohort was established with baseline assessment at Wave 3 (2015). Participants who reported stroke at Waves 1–3 were excluded. The follow-up extended from Wave 3 to the occurrence of stroke or the final survey wave (Wave 5, 2020), whichever occurred first. Stroke events occurring between Waves 4 and 5 were considered outcomes in this analysis. CHARLS investigators instituted robust data quality protocols ensuring accuracy and reliability throughout the prospective surveillance period (20). This self-reported physician-diagnosed stroke ascertainment approach has been extensively utilized in prior CHARLS-based epidemiological research (27,28,34). However, we acknowledge that self-reported stroke may be subject to misclassification, as participants may not accurately recall or differentiate stroke diagnoses from other neurological conditions.

### Assessment of potential confounders

Potential confounding variables were systematically selected and categorized based on established associations with stroke risk and their theoretical relevance to the study framework. Covariates were grouped into sociodemographic characteristics, clinical measurements, lifestyle behaviors, and comorbid conditions. Sociodemographic characteristics included age (continuous variable, measured in years), sex (male or female), educational level (categorized as primary school or below vs secondary school and above), residential location (urban vs rural), and marital status (married vs unmarried). Health insurance status was also recorded as a binary variable. Lifestyle factors comprised smoking status and alcohol consumption, both recorded as binary variables (yes or no). Clinical measurements encompassed blood pressure parameters and biochemical indicators. Blood pressure was assessed through systolic blood pressure (SBP) and diastolic blood pressure (DBP), both measured in mmHg. Laboratory assessments included fasting blood glucose (measured in mg/dL), glycated hemoglobin (HbA1c, expressed as percentage), total cholesterol (mg/dL), low-density lipoprotein cholesterol (LDL-C, mg/dL), and estimated glomerular filtration rate (eGFR, mL/min/1.73 m²). Comorbid conditions were ascertained through self-reported physician diagnosis and defined according to standardized criteria. Participants were classified as hypertensive based on: SBP ≥140 mmHg, DBP ≥90 mmHg, self-declared hypertension diagnosis, or antihypertensive medication intake. Diabetes identification utilized fasting glucose ≥126 mg/dL, glycated hemoglobin ≥6.5%, participant-reported diagnosis, or current diabetes pharmacotherapy. Additional health conditions—including malignancies, chronic lung pathology, liver disorders, and kidney disease—were ascertained via self-reported physician confirmation. Statin or other lipid-lowering drug use was concurrently recorded. All baseline covariate data were collected during the initial survey wave through standardized questionnaires administered by trained interviewers, and anthropometric and laboratory measurements were conducted using validated protocols to ensure data quality and consistency.

### Statistical analysis

R software (version 4.3.2; R Foundation for Statistical Computing, Vienna, Austria) was employed for all statistical procedures. A significance level of P < 0.05 (two-sided) was applied throughout unless otherwise indicated. Parametric continuous variables were described using mean ± SD, whereas non-parametric variables utilized median (IQR). Categorical measures were summarized through frequencies and percentages. Between-group analyses applied t-tests (with Welch correction for unequal variances) for normally distributed continuous data, Mann-Whitney U tests for skewed distributions, and Pearson chi-square tests (substituting Fisher’s exact when expected frequencies <5) for categorical comparisons. Multivariable logistic regression models were constructed to estimate the association between CVAI-FI (both baseline and cumulative) and incident stroke, with results presented as odds ratios (ORs) and 95% confidence intervals (CIs). While Cox regression is conventionally preferred for time-to-event analyses, the binary characterization of incident stroke within a defined observation window justifies the use of logistic regression as a statistically sound and methodologically defensible framework (35,36). In the CHARLS cohort, stroke events were ascertained at discrete survey waves through structured questionnaires, and the observation period was well-defined for all participants. Under these conditions, logistic regression yields valid odds ratio estimates and has been extensively employed in CHARLS-based epidemiological research. Three hierarchical models were fitted: Model 1 was unadjusted; Model 2 adjusted for demographic and lifestyle factors including sex, age, residence (urban/rural), education level (primary school/secondary school and above), marital status (married/unmarried), smoking status (never/ever), drinking status (never/ever), insurance status (yes/no), SBP—selected as the sole blood pressure measure given its stronger and more linear association with stroke risk compared with DBP (37); Model 3 (fully adjusted) further adjusted for metabolic and clinical parameters including HbA1c, FBG, diabetes, cancer, liver disease, kidney disease, chronic lung disease, TC, LDL-C, eGFR, and lipid-lowering medication use. We analyzed CVAI-FI as both a continuous measure (per 1-SD unit increase) and as a categorical variable partitioned by quartiles. Trends across ordered quartile categories were tested by incorporating each quartile’s median value as a continuous predictor in regression models.

To investigate dose-dependent associations and potential nonlinearity, we conducted RCS analyses using four knots placed at the 5th, 35th, 65th, and 95th percentiles of the CVAI-FI distribution. The RCS models were fitted using the same three hierarchical adjustment strategies as the logistic regression models: Model 1 with none covariate adjustment, Model 2 adjusted for demographic and lifestyle factors, and Model 3 with full adjustment for all covariates. All RCS models utilized the median CVAI-FI as the referent value. The overall association was tested using the Wald test, and nonlinearity was evaluated by testing whether the second and third spline term coefficients jointly equaled zero via likelihood ratio testing. Stratified analyses were performed to explore potential heterogeneity of effects across predefined demographic, behavioral, and clinical variables: age (<60 vs. ≥60 years), sex (male vs. female), education level (primary school vs. secondary school and above), smoking status (never vs. ever), marital status (married vs. unmarried), drinking status (never vs. ever), diabetes (no vs. yes), hypertension (no vs. yes), chronic lung disease (no vs. yes), liver disease (no vs. yes), kidney disease (no vs. yes), and residence (urban vs. rural). We examined potential effect modification by incorporating product terms between CVAI-FI and stratification factors into the comprehensive adjustment models, using Wald statistics to assess the significance of these interactions.

We conducted ROC curve analysis to evaluate the predictive performance of CVAI-FI relative to its constituent elements (CVAI and FI separately). The area under the curve (AUC) and 95% CI were calculated for each predictor. Pairwise comparisons of AUCs were conducted using DeLong’s test. To quantify the incremental predictive value provided by CVAI-FI beyond individual components and clinical risk factors, we calculated the net reclassification improvement (NRI) and integrated discrimination improvement (IDI). Missing data analysis revealed that drinking status had 26% missing values, while all other variables had missing rates below 1% (Supplementary Figure S1). Given that drinking is an established risk factor for stroke, missing drinking information was handled through KNN imputation methodology instead of removing individuals with incomplete data. To maximize analytical power and mitigate selection bias, primary analyses relied on imputed rather than complete-case data.

To distinguish the composite index approach from formal statistical interaction testing, we conducted a supplementary analysis in which CVAI (per SD), FI (per SD), and their product term (CVAI × FI) were entered as separate independent variables in the fully adjusted logistic regression model. Model fit was compared using the Akaike Information Criterion (AIC) and likelihood ratio tests (LRT). The incremental predictive value of the interaction term beyond main effects was evaluated using the LRT comparing the main-effects-only model (CVAI + FI) with the interaction model (CVAI + FI + CVAI × FI). The discriminative performance of different model specifications was compared using the area under the receiver operating characteristic curve (AUC) with DeLong’s test.

We conducted several sensitivity analyses to verify the stability of our main results. The first sensitivity analysis employed a complete-case approach, where logistic models were re-fitted exclusively among participants presenting full covariate information without any missing observations. Second, individuals developing stroke within the initial two-year follow-up period (Wave 2) were excluded to reduce potential reverse causality bias, wherein subclinical disease or early pathological changes might have influenced baseline CVAI-FI measurements. Third, given that underlying conditions such as hypertension and diabetes mellitus are documented independent stroke predictors, we conducted separate sensitivity analyses excluding participants with prevalent hypertension, prevalent diabetes at baseline to evaluate whether these conditions confounded the observed associations. Fourth, to address potential residual confounding from drinking status, models excluding alcohol consumption as a covariate were fitted. Fifth, as an alternative analytical strategy, we employed Cox proportional hazards modeling with stroke incidence as the event outcome and observation time as the temporal variable. Non-event participants were right-censored at their final follow-up assessment, whereas participants experiencing stroke were censored at first event occurrence. Findings were reported as hazard ratios (HRs) with 95% confidence intervals. The proportional hazards assumption was confirmed through Schoenfeld residual diagnostic testing. Sixth, survival curves stratified by CVAI-FI quartile categories were constructed using the Kaplan-Meier method, with log-rank tests employed to compare stroke-free survival across strata. To quantify the potential impact of unmeasured confounding, *E*-values were calculated for the fully adjusted Model 3. *E*-values specify the minimal association strength an unmeasured factor would require with both exposure and outcome to fully account for the observed relationship, after covariate adjustment. Greater *E*-values demonstrate superior stability against unmeasured confounding influences.

## Results

### Baseline characteristics of participants

Using CHARLS data, we established two prospective cohorts to evaluate the relationship between both baseline and cumulative CVAI-FI measures and incident stroke. The baseline CVAI-FI analysis included 6,772 participants, whereas the cumulative CVAI-FI analysis comprised 4,170 participants. Tables 1 and 2 present baseline demographic and clinical characteristics according to CVAI-FI quartile distribution. Detailed comparisons between the analytic sample and excluded individuals are documented in Supplementary Materials (Table S6 and Table S7).

**Table 1:** Baseline characteristics of individuals classified by quartiles of the Chinese visceral adiposity index-frailty index (CVAI-FI) among individuals with CKM syndrome stages 0–3.


CHARACTERISTIC	OVERALL (N = 6,772)^1^	Q1 (N = 1,693)^1^	Q2 (N = 1,693)^1^	Q3 (N = 1,693)^1^	Q4 (N = 1,693)^1^	P VALUE^2^

FI index	0.15 ± (0.11)	0.05 ± (0.04)	0.10 ± (0.05)	0.16 ± (0.06)	0.28 ± (0.11)	<0.001

Frailty	1,049 (15%)	10 (0.6%)	24 (1.4%)	136 (8.0%)	879 (52%)	<0.001

CKM						<0.001

0	280 (4.1%)	134 (7.9%)	79 (4.7%)	48 (2.8%)	19 (1.1%)	

1	749 (11%)	235 (14%)	209 (12%)	187 (11%)	118 (7.0%)	

2	1,573 (23%)	368 (22%)	388 (23%)	448 (26%)	369 (22%)	

3	4,170 (62%)	956 (56%)	1,017 (60%)	1,010 (60%)	1,187 (70%)	

Age, years	59 ± (9)	55 ± (8)	57 ± (9)	59 ± (9)	64 ± (10)	<0.001

Sex, male	3,198 (47%)	982 (58%)	891 (53%)	739 (44%)	586 (35%)	<0.001

Education level	2,054 (30%)	771 (46%)	602 (36%)	420 (25%)	261 (15%)	<0.001

Smoking	2,649 (39%)	772 (46%)	731 (43%)	625 (37%)	521 (31%)	<0.001

Marital status (married)	5,996 (89%)	1,567 (93%)	1,535 (91%)	1,492 (88%)	1,402 (83%)	<0.001

Drinking	1,291 (19%)	241 (14%)	336 (20%)	328 (19%)	386 (23%)	<0.001

Glucose (mg/dl)	109 ± (34)	105 ± (29)	106 ± (27)	109 ± (32)	116 ± (44)	<0.001

Glycated Hemoglobin (%)	5.26 ± (0.81)	5.16 ± (0.74)	5.20 ± (0.69)	5.27 ± (0.79)	5.43 ± (0.96)	<0.001

Total Cholesterol (mg/dl)	194 ± (38)	190 ± (38)	192 ± (38)	194 ± (36)	200 ± (40)	<0.001

LDL Cholesterol (mg/dl)	117 ± (35)	115 ± (34)	116 ± (34)	117 ± (34)	120 ± (37)	<0.001

eGFR, mL/min/1.73 m^2^	93 ± (14)	96 ± (13)	94 ± (14)	93 ± (14)	89 ± (15)	<0.001

SBP, mmHg	129 ± (21)	123 ± (18)	126 ± (20)	129 ± (21)	136 ± (23)	<0.001

DBP, mmHg	75 ± (12)	74 ± (12)	74 ± (12)	75 ± (12)	77 ± (12)	<0.001

Hypertension	2,508 (37%)	344 (20%)	536 (32%)	656 (39%)	972 (57%)	<0.001

Diabetes	1,015 (15%)	157 (9.3%)	195 (12%)	258 (15%)	405 (24%)	<0.001

Cancer	55 (0.8%)	9 (0.5%)	9 (0.5%)	17 (1.0%)	20 (1.2%)	0.074

Chronic lung disease	610 (9.0%)	60 (3.5%)	111 (6.6%)	182 (11%)	257 (15%)	<0.001

Liver disease	249 (3.7%)	32 (1.9%)	56 (3.3%)	71 (4.2%)	90 (5.3%)	<0.001

Kidney disease	398 (5.9%)	80 (4.7%)	87 (5.1%)	106 (6.3%)	125 (7.4%)	0.004

Lipid-lowering drugs	247 (3.6%)	21 (1.2%)	44 (2.6%)	70 (4.1%)	112 (6.6%)	<0.001

Insurance	6,401 (95%)	1,601 (95%)	1,610 (95%)	1,592 (94%)	1,598 (94%)	0.6

Residence (urban)	3,880 (57%)	1,024 (60%)	994 (59%)	976 (58%)	886 (52%)	<0.001


^1^Mean ± (SD); n (%); ^2^Kruskal-Wallis rank sum test; Pearson’s Chi-squared test.

**Table 2:** Baseline characteristics of individuals classified by quartiles of the cumulative Chinese visceral adiposity index-frailty index (CVAI-FI) among individuals with CKM syndrome stages 0–3.


CHARACTERISTIC	OVERALL (N = 4,170)^1^	Q1 (N =1,043)^1^	Q2 (N =1,042)^1^	Q3 (N =1,042)^1^	Q4 (N =1,043)^1^	P VALUE^2^

FI index_2011_	0.14 ± (0.11)	0.06 ± (0.05)	0.11 ± (0.06)	0.15 ± (0.08)	0.25 ± (0.12)	<0.001

FI index_2015_	0.18 ± (0.13)	0.07 ± (0.05)	0.13 ± (0.07)	0.19 ± (0.08)	0.32 ± (0.13)	<0.001

Frailty_2011_	591 (14%)	8 (0.8%)	30 (2.9%)	109 (10%)	444 (43%)	<0.001

Frailty_2015_	939 (23%)	7 (0.7%)	46 (4.4%)	200 (19%)	686 (66%)	<0.001

CKM						<0.001

0	172 (4.1%)	76 (7.3%)	44 (4.2%)	43 (4.1%)	9 (0.9%)	

1	509 (12%)	149 (14%)	153 (15%)	125 (12%)	82 (7.9%)	

2	1,029 (25%)	217 (21%)	263 (25%)	280 (27%)	269 (26%)	

3	2,460 (59%)	601 (58%)	582 (56%)	594 (57%)	683 (65%)	

Age, years	58 ± (9)	55 ± (8)	57 ± (8)	59 ± (8)	62 ± (9)	<0.001

Sex, male	1,901 (46%)	637 (61%)	531 (51%)	422 (40%)	311 (30%)	<0.001

Education level	1,285 (31%)	498 (48%)	353 (34%)	265 (25%)	169 (16%)	<0.001

Smoking	1,570 (38%)	496 (48%)	439 (42%)	356 (34%)	279 (27%)	<0.001

Marital status (married)	3,756 (90%)	978 (94%)	960 (92%)	928 (89%)	890 (85%)	<0.001

Drinking	790 (19%)	173 (17%)	198 (19%)	197 (19%)	222 (21%)	0.058

Glucose (mg/dl)	109 ± (33)	104 ± (24)	106 ± (26)	110 ± (37)	115 ± (41)	<0.001

Glycated Hemoglobin (%)	5.26 ± (0.79)	5.13 ± (0.61)	5.17 ± (0.70)	5.31 ± (0.82)	5.43 ± (0.96)	<0.001

Total Cholesterol (mg/dl)	194 ± (38)	188 ± (36)	193 ± (38)	195 ± (37)	200 ± (39)	<0.001

LDL Cholesterol (mg/dl)	117 ± (35)	112 ± (32)	117 ± (35)	118 ± (35)	121 ± (36)	<0.001

eGFR	94 ± (14)	96 ± (13)	95 ± (13)	94 ± (14)	90 ± (15)	<0.001

SBP, mmHg	128 ± (20)	122 ± (17)	125 ± (19)	129 ± (20)	135 ± (22)	<0.001

DBP, mmHg	75 ± (12)	73 ± (11)	74 ± (12)	75 ± (12)	77 ± (12)	<0.001

Hypertension	1,503 (36%)	200 (19%)	295 (28%)	426 (41%)	582 (56%)	<0.001

Diabetes	618 (15%)	84 (8.1%)	117 (11%)	171 (16%)	246 (24%)	<0.001

Cancer	36 (0.9%)	4 (0.4%)	8 (0.8%)	11 (1.1%)	13 (1.2%)	0.2

Chronic lung disease	372 (8.9%)	41 (3.9%)	69 (6.6%)	103 (9.9%)	159 (15%)	<0.001

Liver disease	137 (3.3%)	24 (2.3%)	34 (3.3%)	39 (3.7%)	40 (3.8%)	0.2

Kidney disease	221 (5.3%)	44 (4.2%)	46 (4.4%)	58 (5.6%)	73 (7.0%)	0.017

Lipid-lowering drugs	171 (4.1%)	17 (1.6%)	29 (2.8%)	41 (3.9%)	84 (8.1%)	<0.001

Insurance	3,968 (95%)	997 (96%)	995 (95%)	992 (95%)	984 (94%)	0.5

Residence (urban)	2,465 (59%)	647 (62%)	637 (61%)	592 (57%)	589 (56%)	0.013


^1^Mean ± (SD); n (%); ^2^Kruskal-Wallis rank sum test; Pearson’s Chi-squared test.

Higher CVAI-FI quartile groups demonstrated older age distributions, with a larger percentage of participants ≥60 years. Sex distribution varied significantly across quartiles, with higher proportions of females in Q2 and Q3. Educational level and marital status also showed significant differences among quartiles, while ethnicity distribution remained comparable across groups. Key metabolic parameters, including BMI, WC, TG, and FPG, demonstrated progressive increases from Q1 to Q4 (all *P <* 0.001). Similarly, TC, CRP, and HbA1c levels were significantly elevated in higher quartiles. The prevalence of hypertension, dyslipidemia, diabetes, heart disease, stroke, and kidney disease also increased progressively from Q1 to Q4 (all *P <* 0.001) (Table 1). The cumulative CVAI-FI assessment revealed consistent findings (Table 2). These findings suggest that elevated baseline and cumulative CVAI-FI values were associated with older age, adverse metabolic profiles, and increased burden of cardiometabolic risk factors and comorbidities at baseline.

### Association of baseline CVAI-FI and cumulative CVAI-FI with new-onset stroke in CKM stage 0–3

In the fully adjusted model including CVAI, FI, and the CVAI × FI product term as separate variables, both CVAI (OR = 1.29 per SD, 95% CI 1.16–1.44, *P <* 0.001) and FI (OR = 1.28 per SD, 95% CI 1.15–1.41, *P <* 0.001) remained independently associated with incident stroke. However, the multiplicative interaction term was not statistically significant (OR = 0.96, 95% CI 0.88–1.04, P = 0.312). The likelihood ratio test confirmed that adding the interaction term did not significantly improve model fit beyond the main-effects-only model (χ² = 1.02, *P* = 0.312). In contrast, adding FI to a CVAI-only model (χ² = 20.74, *P <* 0.001) and adding CVAI to an FI-only model (χ² = 21.02, *P <* 0.001) both yielded statistically significant improvements in model fit. Among the model specifications examined, the main-effects-only model (CVAI + FI) achieved the lowest AIC (3129.34) and a comparable AUC (0.706) to the interaction model (AIC = 3130.32; AUC = 0.706; DeLong *P* = 0.559), while the CVAI-FI composite index demonstrated an AUC of 0.702 (Supplementary Table S2). These findings indicate that CVAI and FI contribute complementary additive—rather than multiplicative synergistic—information for stroke risk prediction, supporting the utility of the CVAI-FI composite as a practical integrated risk marker.

Incident stroke occurred in 447 participants (baseline cohort) and 258 participants (cumulative cohort) during prospective follow-up. Both baseline and cumulative CVAI-FI demonstrated statistically significant positive associations with stroke incidence across all regression models (Tables 3 and 4). For baseline CVAI-FI, when compared to the lowest quartile (Q1), those in Q4 showed a statistically significant 177% higher stroke risk after full covariate adjustment (Model 3: OR = 2.77, 95% CI = 1.96–3.96, *P <* 0.001). The associations remained robust and showed a clear dose-response relationship across quartiles (Q2: OR = 1.71, 95% CI = 1.21–2.44, *P <* 0.001; Q3: OR = 2.10, 95% CI = 1.50–2.99, *P <* 0.001). Progressive adjustment for demographic factors (Model 1), lifestyle variables and metabolic parameters (Model 2), and chronic diseases (Model 3) did not substantially attenuate the associations, with all *P* values for trend being statistically significant (*P <* 0.001) (Table 3). Similarly, for cumulative CVAI-FI, the highest quartile (Q4) was associated with a significantly elevated stroke risk compared with Q1 in the fully adjusted model (Model 3: OR = 4.55, 95% CI = 2.83–7.57, *P <* 0.001). The dose-response relationship was apparent across quartiles (Q2: OR = 1.98, 95% CI = 1.22–3.33, *P <* 0.001; Q3: OR = 2.16, 95% CI = 1.32–3.62, *P <* 0.001), with consistently significant trends across all adjustment models (all *P* for trend <0.001) (Table 4).

**Table 3:** Association between the Chinese visceral adiposity index (CVAI)-frailty index and stroke incidence in individuals with Cardiovascular-Kidney-Metabolic Syndrome Stage 0–3.


CHARACTERISTIC	MODEL 1 OR (95%CI), P	MODEL 2 OR (95%CI), P	MODEL 3 OR (95%CI), P

Per unit increase	1.027 (1.022, 1.033), <0.0001	1.022 (1.016, 1.028), <0.0001	1.020 (1.013, 1.026), <0.0001

Per SD increase	1.446 (1.343, 1.555), <0.0001	1.346 (1.237, 1.463), <0.0001	1.312 (1.201, 1.432), <0.0001

CVAI-FI quartile			

Q1	1 (Reference)	1 (Reference)	1 (Reference)

Q2	1.855 (1.319, 2.637)**	1.742 (1.234, 2.485)**	1.706 (1.208, 2.435)**

Q3	2.407 (1.737, 3.383)**	2.197 (1.570, 3.113)**	2.103 (1.499, 2.986)**

Q4	3.778 (2.775, 5.230)**	3.040 (2.172, 4.314)**	2.772 (1.965, 3.963)**

P for trend	<0.0001	<0.0001	<0.0001


Model 1: unadjusted for any covariates; Model 2: adjusted for gender, age, residence, education level, marital status, smoking status, drinking status, insurance status, and systolic blood pressure (SBP); Model 3: adjusted for gender, age, residence, education level, marital status, smoking status, drinking status, insurance status, systolic blood pressure (SBP), Glycated hemoglobin (HBA1c), fasting blood glucose (FBG), diabetes, cancer, liver cancer, kidney disease, chronic lung disease, total cholesterol (TC), low-density lipoprotein cholesterol (LDL-C), estimated glomerular filtration rate (eGFR), and lipid-lowering drugs. Note: *P < 0.05, **P < 0.01.

**Table 4:** Association between the cumulative Chinese visceral adiposity index-frailty index (CVAI-FI) and stroke incidence in individuals with Cardiovascular-Kidney-Metabolic Syndrome Stage 0–3.


CHARACTERISTIC	MODEL 1 OR (95%CI), P	MODEL 2 OR (95%CI), P	MODEL 3 OR (95%CI), P

Per unit increase	1.012 (1.010, 1.015), <0.0001	1.011 (1.008, 1.013), <0.0001	1.011 (1.008, 1.014), <0.0001

Per SD increase	1.637 (1.485, 1.802), <0.0001	1.542 (1.380, 1.720), <0.0001	1.536 (1.366, 1.724), <0.0001

Cumulative CVAI-FI quartile			

Q1	1 (Reference)	1 (Reference)	1 (Reference)

Q2	2.140 (1.320, 3.565)**	2.036 (1.249, 3.407)**	1.984 (1.216, 3.325)**

Q3	2.503 (1.563, 4.131)**	2.238 (1.377, 3.743)**	2.159 (1.323, 3.623)**

Q4	5.834 (3.808, 9.319)**	4.838 (3.041, 7.977)**	4.548 (2.825, 7.572)**

P for trend	<0.0001	<0.0001	<0.0001


Model 1: unadjusted for any covariates; Model 2: adjusted for gender, age, residence, education level, marital status, smoking status, drinking status, insurance status, and systolic blood pressure (SBP); Model 3: adjusted for gender, age, residence, education level, marital status, smoking status, drinking status, insurance status, systolic blood pressure (SBP), diastolic blood pressure (DBP), Glycated hemoglobin (HBA1c), fasting blood glucose (FBG), diabetes, cancer, liver cancer, kidney disease, chronic lung disease, total cholesterol (TC), low-density lipoprotein cholesterol (LDL-C), estimated glomerular filtration rate (eGFR), and lipid-lowering drugs. Note: *P < 0.05, **P < 0.01.

### Dose-response relationship between baseline CVAI-FI and cumulative CVAI-FI with new-onset stroke in CKM stage 0–3

To characterize nonlinear exposure-outcome relationships, RCS modeling assessed associations between CVAI-FI measures and stroke incidence. As shown in Figure 2, baseline CVAI-FI demonstrated significant nonlinear associations with incident stroke across all three analytical models. Model 1 (crude) showed strong overall statistical significance (*P* for overall < 0.001) with clear evidence of nonlinearity (*P* for nonlinear < 0.001). After controlling for demographic and behavioral factors (Model 2), the nonlinear relationship persisted (*P* for nonlinear < 0.001). Complete adjustment (Model 3) confirmed the robustness of this nonlinear pattern (*P* for nonlinear <0.001), with stroke risk accelerating disproportionately at higher CVAI-FI levels, suggesting threshold effects beyond which metabolic burden translates into markedly elevated stroke risk. Similar nonlinear patterns were observed for cumulative CVAI-FI (Figure 3). The unadjusted model revealed a statistically significant overall relationship (*P* for overall < 0.001) with evidence of curvature (*P* for nonlinear = 0.008). Progressive adjustments in Model 2 (*P* for overall < 0.001; *P* for nonlinear = 0.006) and Model 3 (*P* for overall < 0.001; *P* for nonlinear = 0.034) confirmed the persistence of the nonlinear relationship. Notably, the cumulative CVAI-FI exhibited steeper risk escalation at higher values compared to baseline measurements, indicating that sustained exposure to elevated visceral adiposity and frailty burden confers greater stroke risk than single-point assessments. Restricted cubic spline analyses revealed a nonlinear dose-response relationship, with stroke risk increasing more steeply once baseline CVAI-FI exceeded approximately 30 (OR >1.2) and once cumulative CVAI-FI exceeded approximately 55 (OR >1.2). At the upper end of the distribution, participants in the highest quartile exhibited substantially elevated stroke risk (baseline CVAI-FI Q4: OR = 1.94; cumulative CVAI-FI Q4: OR = 2.07).

**Figure 2:**
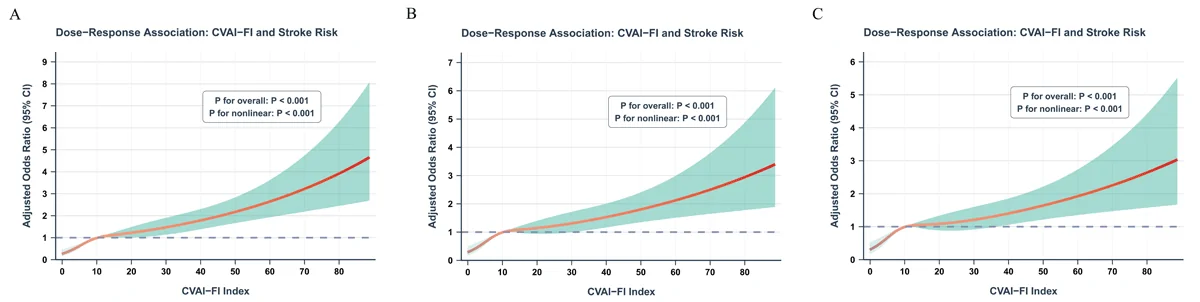
Restricted cubic spline analysis of the dose-response relationship between baseline CVAI-FI and stroke incidence. **(A)** Model 1: crude model. **(B)** Model 2: adjusted for demographic and lifestyle factors (gender, age, residence, education, marital status, smoking, drinking, insurance, SBP). **(C)** Model 3: fully adjusted model including Model 2 covariates plus metabolic and clinical parameters (HbA1c, FBG, diabetes, cancer, liver disease, kidney disease, chronic lung disease, TC, LDL-C, eGFR, lipid-lowering medication). The solid red line represents adjusted odds ratio with shaded area indicating 95% CI. Knots were placed at the 5th, 35th, 65th, and 95th percentiles. All models show significant nonlinear associations (*P* for overall < 0.001; *P* for nonlinear < 0.001).

**Figure 3:**
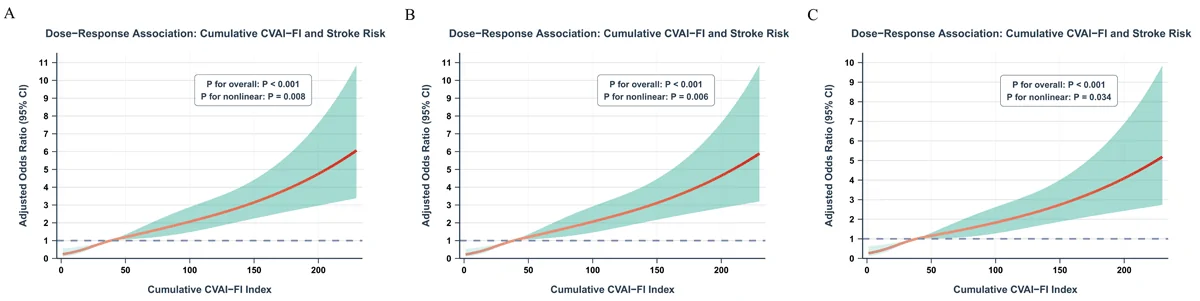
Restricted cubic spline analysis of the dose-response relationship between cumulative CVAI-FI and stroke incidence. **(A)** Model 1: crude model. **(B)** Model 2: adjusted for demographic and lifestyle factors (gender, age, residence, education, marital status, smoking, drinking, insurance, SBP). **(C)** Model 3: fully adjusted model including Model 2 covariates plus metabolic and clinical parameters (HbA1c, FBG, diabetes, cancer, liver disease, kidney disease, chronic lung disease, TC, LDL-C, eGFR, lipid-lowering medication). The solid red line represents adjusted odds ratio with shaded area indicating 95% CI. All models show significant nonlinear associations (*P* for overall < 0.001; *P* for nonlinear < 0.01).

### Subgroup analysis and interaction tests

Stratified analyses across predefined demographic, behavioral, and clinical subgroups evaluated the stability of associations and examined potential effect heterogeneity. The results are summarized in forest plots for both baseline CVAI-FI (Figure 4) and cumulative CVAI-FI (Figure 5). For baseline CVAI-FI, positive associations between elevated CVAI-FI levels and increased stroke risk were consistently observed across all predefined subgroups. The point estimates of the ORs and their 95% CIs demonstrated generally homogeneous effects across most stratification variables. Formal tests for interaction revealed statistically significant effect modification by education level (*P* for interaction < 0.001), marital status (*P* for interaction = 0.019), and hypertension status (*P* for interaction = 0.0019). Specifically, the association was stronger among participants with secondary school education and above (OR = 1.828, 95% CI = 1.494–2.236) compared to those with primary school education (OR = 1.200, 95% CI = 1.082–1.326). Similarly, participants without hypertension showed a more pronounced association (OR = 1.509, 95% CI = 1.299–1.747) compared to those with hypertension (OR = 1.199, 95% CI = 1.071–1.338). No significant interactions were observed for age (*P* = 0.055), sex (*P* = 0.578), smoking status (*P* = 0.625), drinking status (*P* = 0.66), diabetes (*P* = 0.608), chronic lung disease (*P* = 0.1), liver disease (*P* = 0.955), kidney disease (*P* = 0.258), or residence (*P* = 0.842). Similar patterns were observed for cumulative CVAI-FI. The observed relationships persisted across all subpopulations, with significant effect modifications detected for age (*P* for interaction = 0.033), education level (*P* for interaction = 0.0042), and marital status (*P* for interaction = 0.046). The effect appeared stronger in younger participants aged <60 years (OR = 1.841, 95% CI = 1.505–2.247) compared to those aged ≥60 years (OR = 1.391, 95% CI = 1.196–1.611). Participants with higher education demonstrated a stronger association (OR = 2.244, 95% CI = 1.708–2.968) than those with primary education (OR = 1.409, 95% CI = 1.230–1.610). No significant interactions were found for sex (*P* = 0.358), smoking status (*P* = 0.906), drinking status (*P* = 0.995), diabetes (*P* = 0.757), hypertension (*P* = 0.7), chronic lung disease (*P* = 0.674), liver disease (*P* = 0.663), kidney disease (*P* = 0.118), or residence (*P* = 0.834). These findings suggest that the identified associations between both baseline and cumulative CVAI-FI and stroke risk are broadly consistent across diverse patient populations, while highlighting education level and marital status as potential modifiers of the risk relationship.

**Figure 4:**
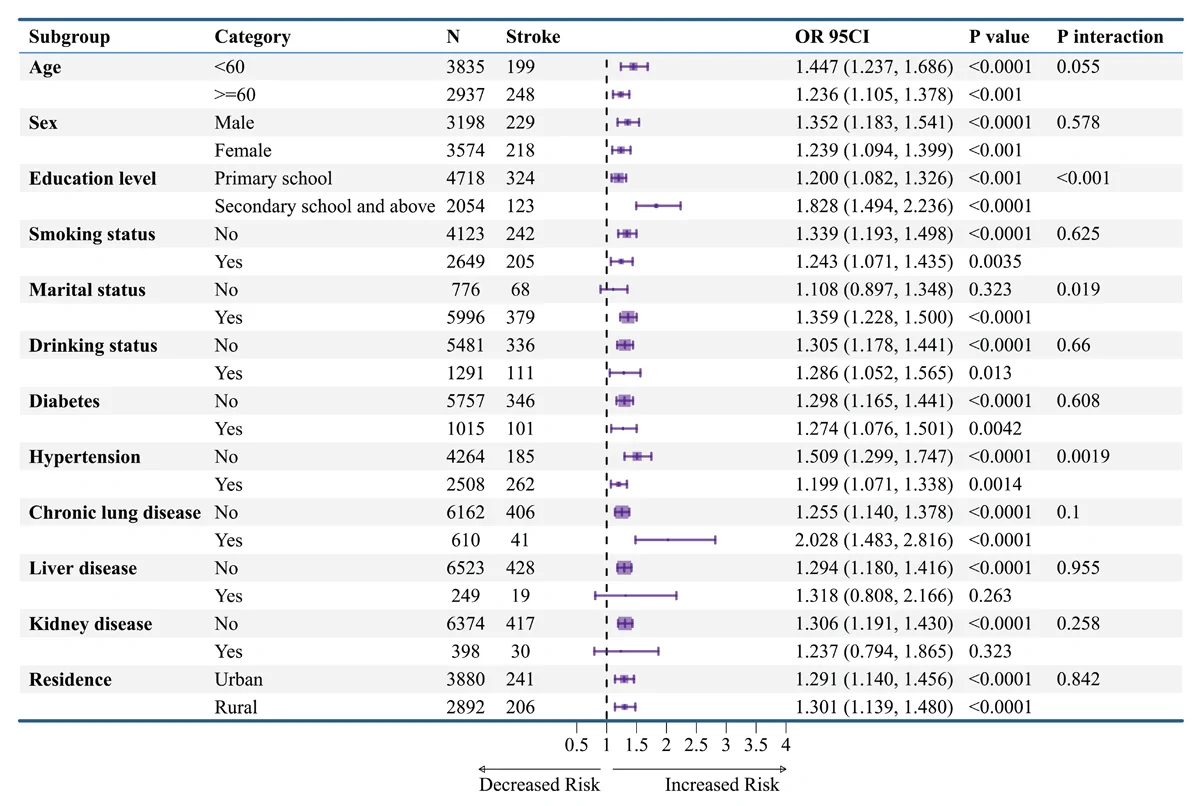
Subgroup analysis of the association between baseline CVAI-FI and stroke incidence. Forest plot presenting subgroup-specific odds ratios (ORs) and 95% confidence intervals (CIs) for the association between baseline CVAI-FI (per 1 standard deviation increase) and incident stroke across pre-specified demographic, lifestyle, and clinical subgroups.

**Figure 5:**
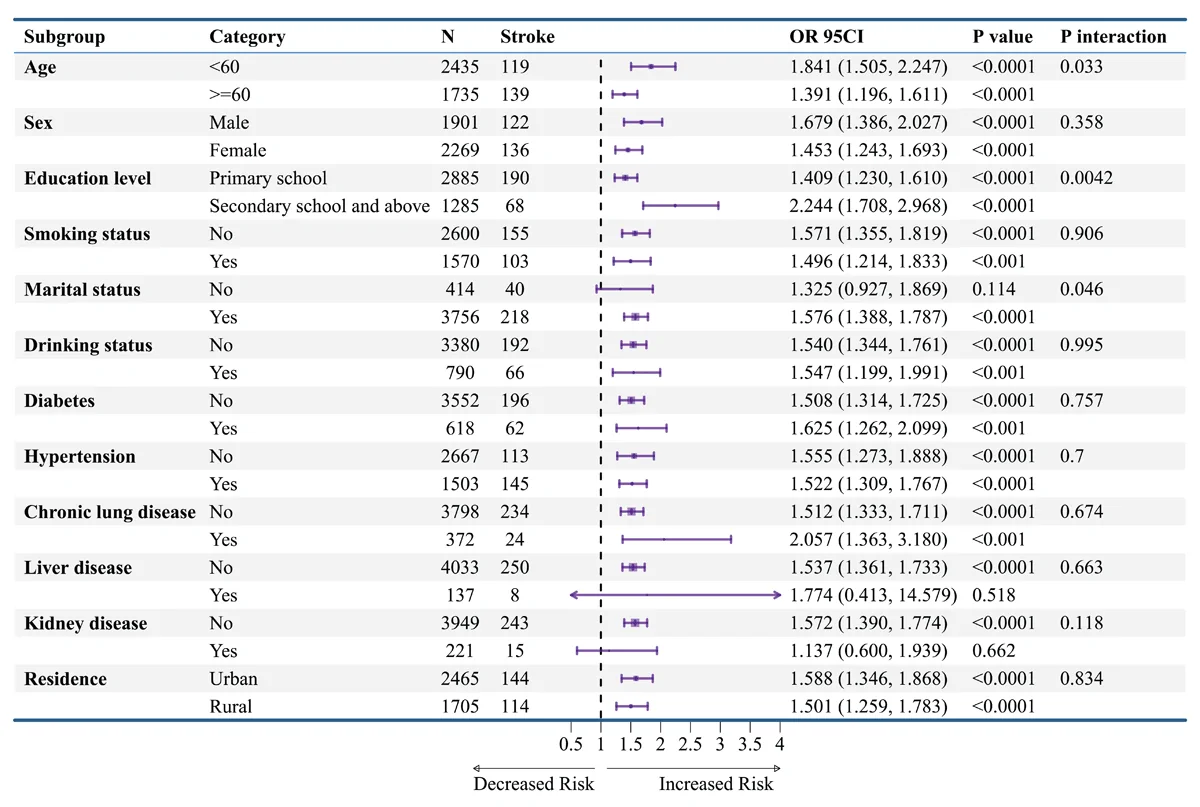
Subgroup analysis of the association between cumulative CVAI-FI and stroke incidence. Forest plot presenting subgroup-specific odds ratios (ORs) and 95% confidence intervals (CIs) for the association between cumulative CVAI-FI (per 1 standard deviation increase) and incident stroke across pre-specified demographic, lifestyle, and clinical subgroups. Cumulative CVAI-FI represents the average of measurements at Wave 1 (2011) and Wave 3 (2015). Black squares represent OR point estimates with size proportional to precision; horizontal lines indicate 95% CIs. The vertical dashed line at OR = 1.0 represents the null value. All models were adjusted for demographics, lifestyle, metabolic, and clinical covariates, with stratification variables excluded from respective analyses. *P* values test within-subgroup associations; *P* for interaction tests heterogeneity across subgroup categories.

### Sensitivity analysis

We executed several sensitivity analyses to verify the consistency of primary results. First, when participants with missing covariate data were removed, associations between CVAI-FI measures (baseline and cumulative) and stroke remained statistically significant across all adjustment models. For baseline CVAI-FI, Q4 participants exhibited markedly increased risk relative to Q1 (Model 3: OR = 2.458, 95% CI = 1.656–3.711, *P <* 0.001; Supplementary Table S8. Similarly, for cumulative CVAI-FI, Q4 demonstrated even stronger associations (Model 3: OR = 3.432, 95% CI = 2.002–6.112, P < 0.001; Supplementary Table S9. To address potential reverse causality, we excluded participants with prevalent stroke at Wave 2 and observed consistent results (Q4 vs. Q1: OR = 2.580, 95% CI = 1.789–3.774, *P <* 0.001; Supplementary Table S10), reinforcing that the observed associations were not driven by early, potentially prevalent cases. Second, recognizing that drinking status had a high proportion of missing data (26.0%), we performed analyses excluding this variable from the adjustment models. The associations remained robust for both baseline CVAI-FI (Q4 vs. Q1: OR = 2.504, 95% CI = 1.691–3.770, *P <* 0.001; Supplementary Table S11) and cumulative CVAI-FI (Q4 vs. Q1: OR = 4.520, 95% CI = 2.797–7.551, *P <* 0.001; Supplementary Table S12 Third, to limit confounding from established cardiovascular risk factors, participants with prevalent hypertension (Supplementary Tables S13 and S14) or diabetes (Supplementary Tables S15 and S16) at baseline were excluded. The positive associations persisted in these disease-free subpopulations. Notably, the effect estimates were stronger among participants without hypertension at baseline (baseline CVAI-FI Q4 vs. Q1: OR = 3.842, 95% CI = 2.366–6.337; cumulative CVAI-FI Q4 vs. Q1: OR = 3.700, 95% CI = 1.883–7.753), suggesting that CVAI-FI may have predictive value in individuals without established hypertension. Fourth, we performed Cox proportional hazards regression analyses as an alternative analytical approach. Kaplan-Meier survival curves demonstrated clear separation across CVAI-FI quartiles (Supplementary Figure S2). Restricted cubic spline analyses using Cox models confirmed significant nonlinear dose-response relationships for both baseline CVAI-FI (Supplementary Figure S3) and cumulative CVAI-FI (Supplementary Figure S4), consistent with our primary logistic regression findings. Subgroup analyses using Cox regression (Supplementary Figures S5 and S6) yielded results comparable to those from logistic models, with the proportional hazards assumption satisfied (Schoenfeld test, all P > 0.05). Finally, to assess the potential impact of unmeasured confounding, we calculated E-values for the primary associations. The *E*-values for one-SD increases were 1.78 (95% CI lower limit: 1.60) for baseline CVAI-FI and 1.53 (95% CI lower limit: 1.40) for cumulative CVAI-FI (Supplementary Figure S7). This indicates an unmeasured confounder must exhibit risk ratios ≥1.78 or ≥1.53 with both exposure and outcome, respectively, independent of controlled covariates, to entirely account for the observed associations, suggesting reasonable robustness against unmeasured confounding. Thus, these sensitivity analyses demonstrated robust and consistent primary results across diverse analytical methods, population subsets, and covariate adjustment strategies. Additionally, given the inherent scale difference between CVAI and FI, we performed sensitivity analyses confirming that alternative formulations—including a sum-based composite—yielded consistent results (Supplementary Table S3, corroborated by published metabolic-frailty indices employing analogous multiplicative approaches (15,29,31,32).

### CVAI-FI performance for stroke prediction

We employed ROC curve techniques to evaluate baseline index performance in stroke risk discrimination (Figure 6). Baseline CVAI-FI demonstrated superior performance (AUC 0.636, 95% CI 0.611–0.662) compared to FI alone (AUC 0.601, 95% CI 0.574–0.627, *P <* 0.001 by DeLong’s test) and comparable performance to CVAI alone (AUC 0.620, 95% CI 0.593–0.647, *P* = 0.222), indicating that the composite index captures complementary dimensions of metabolic and physiological vulnerability. To assess longitudinal exposure effects, we compared baseline measurements, Wave 3 measurements, and cumulative CVAI-FI in 4,170 participants (Figure 7). Cumulative CVAI-FI exhibited the highest discriminative performance (AUC 0.675, 95% CI 0.642–0.709), significantly outperforming baseline CVAI (AUC 0.616, *P <* 0.001), baseline FI (AUC 0.609, *P <* 0.001), baseline CVAI-FI (AUC 0.641, *P <* 0.001), CVAI_2015_ (AUC 0.610, *P <* 0.001), and FI_2015_ (AUC 0.646, *P* = 0.014). Although CVAI-FI_2015_ also exhibited good performance (AUC 0.666, 95% CI 0.632–0.700), it did not significantly differ from cumulative CVAI-FI (*P* = 0.186).

**Figure 6:**
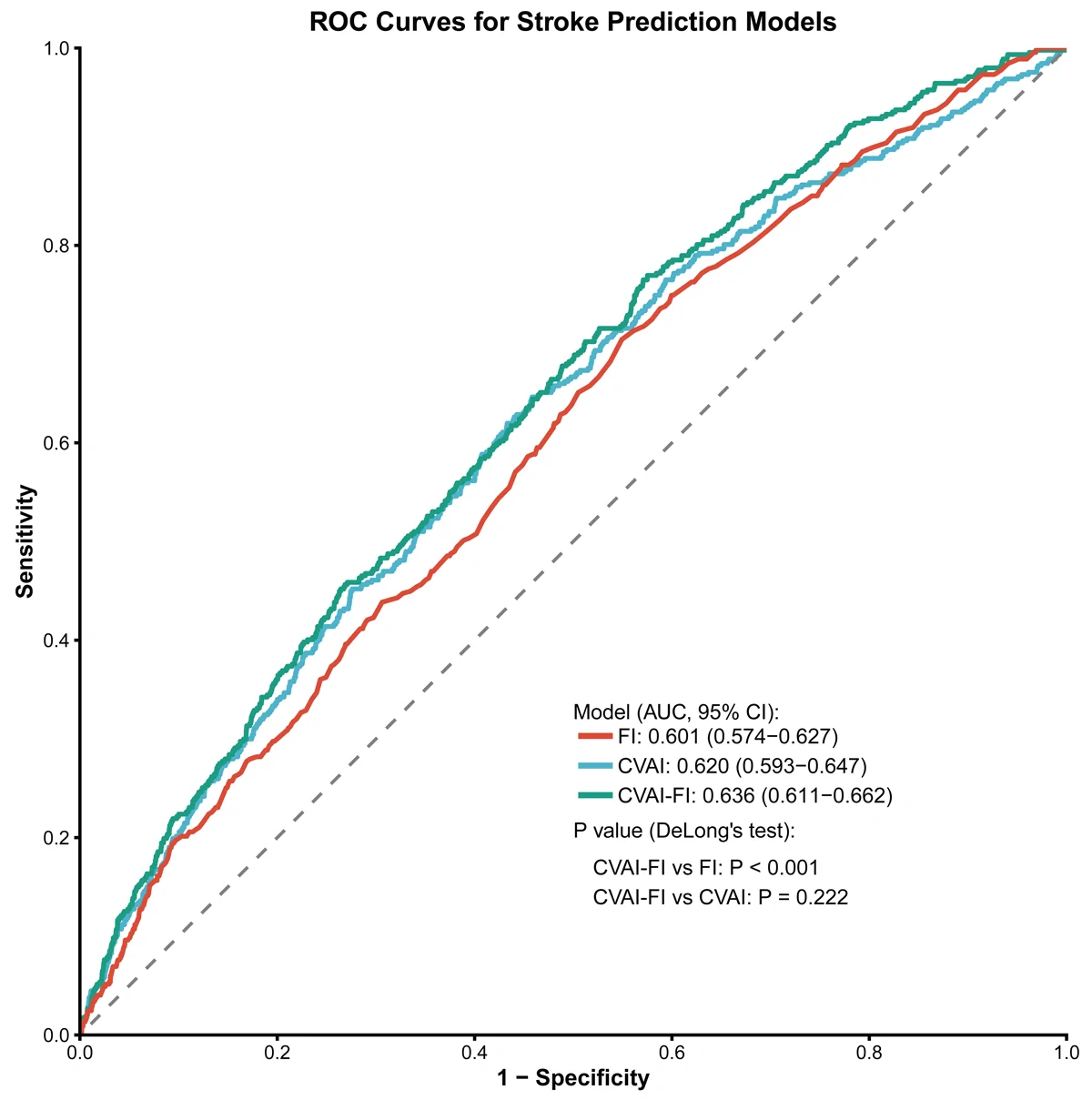
Receiver operating characteristic curves comparing predictive performance of baseline indices for stroke prediction. Receiver operating characteristic (ROC) curves comparing the discriminatory capacity of baseline frailty index (FI), Chinese visceral adiposity index (CVAI), and their combination (CVAI-FI) for predicting incident stroke over 9-year follow-up. The y-axis represents sensitivity (true positive rate) and the x-axis represents 1-specificity (false positive rate). Each colored curve corresponds to one predictive model, with the area under the curve (AUC) and 95% CI displayed in the legend.

**Figure 7:**
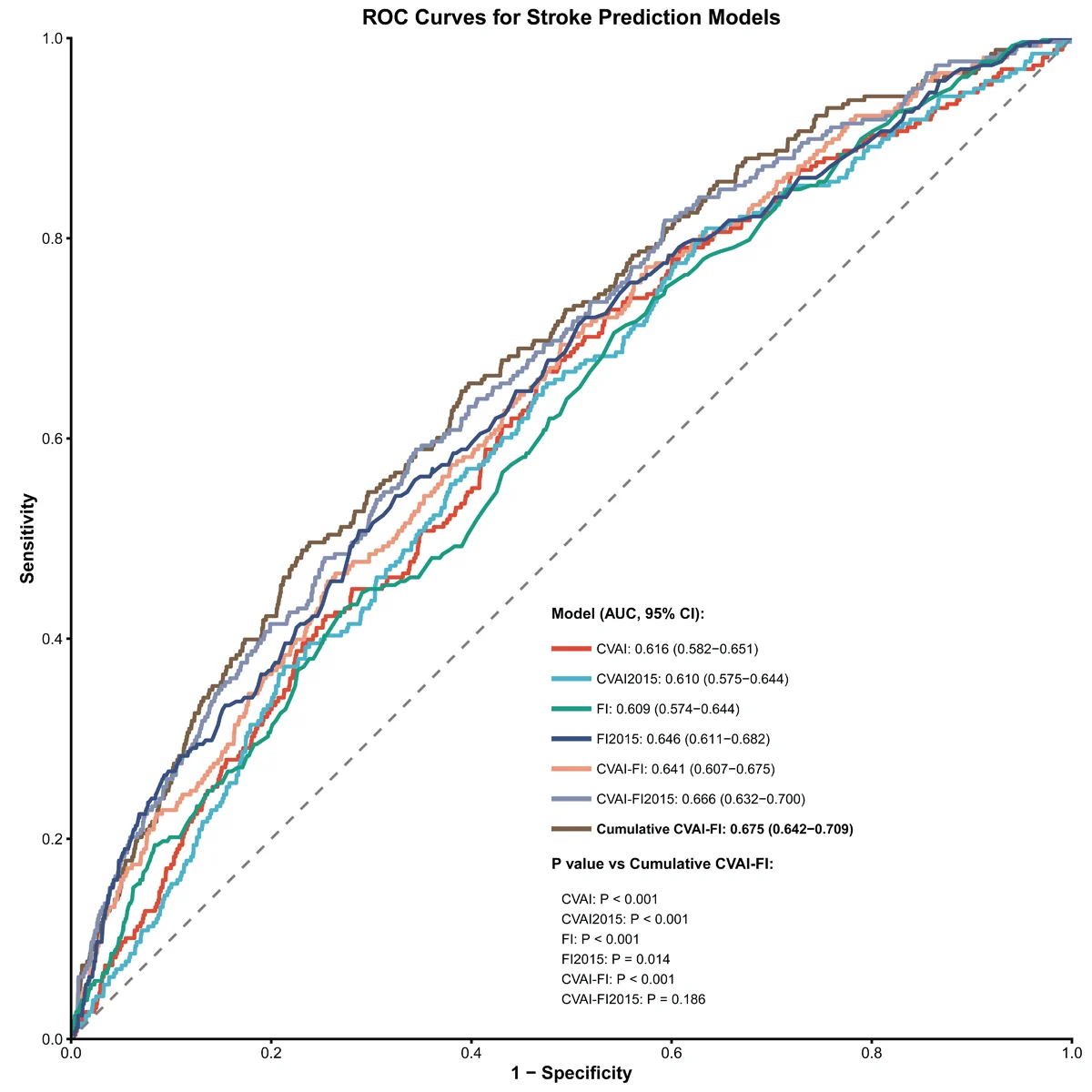
Receiver operating characteristic curves comparing predictive performance of cumulative indices for stroke prediction. Receiver operating characteristic (ROC) curves comparing the discriminatory capacity of multiple baseline and cumulative indices for predicting incident stroke. Models include baseline measurements (Wave 1, 2011): CVAI (AUC = 0.616), FI (AUC = 0.609), CVAI-FI (AUC = 0.641); Wave 3 measurements (2015): CVAI2015 (AUC = 0.610), FI2015 (AUC = 0.646), CVAI-FI2015 (AUC = 0.666); and cumulative CVAI-FI (AUC = 0.675, 95% CI: 0.642–0.709). Each colored curve represents one model, with AUC values and 95% CIs displayed in the legend.

Quantitative assessment of incremental predictive value revealed significant improvements beyond comprehensive clinical models (Supplementary Figure S8 and Figure S9). For baseline CVAI-FI, integrated discrimination improvement (IDI) analysis demonstrated that CVAI-FI provided superior discrimination over the clinical reference model (IDI = 0.005, 95% CI: 0.003–0.007, *P <* 0.001) and showed significant incremental improvement over FI alone (IDI = 0.003, 95% CI: 0.002–0.005, *P <* 0.001) and CVAI alone (IDI = 0.002, 95% CI: 0.001–0.003, *P* = 0.018). Net reclassification improvement (NRI) analysis indicated modest reclassification gains (NRI = 0.032, 95% CI: 0.003–0.061, *P* = 0.029). For cumulative CVAI-FI, substantially greater incremental value was observed, with IDI = 0.020 (95% CI: 0.013–0.026, *P <* 0.001) compared to the clinical model, outperforming CVAI_2015_ (IDI = 0.002, *P >* 0.05), FI_2015_ (IDI = 0.018, *P <* 0.001), and baseline CVAI-FI (IDI = 0.005, *P <* 0.001). Although NRI values did not reach statistical significance (NRI = 0.050, 95% CI: –0.015–0.115, *P* = 0.13), the consistent IDI improvements indicate that cumulative CVAI-FI captures stroke risk dimensions not fully accounted for by traditional clinical variables.

## Discussion

This investigation examines the relationship between the integrated CVAI-FI metric and stroke occurrence among participants with CKM syndrome stages 0–3. Using longitudinal data from 6,772 participants in the CHARLS. Elevated CVAI-FI levels demonstrated a robust, independent, and nonlinear positive association with incident stroke risk in individuals with CKM syndrome stages 0–3, with each standard deviation increase conferring a 29% higher stroke risk after full adjustment. Cumulative CVAI-FI exposure exhibited superior discriminative performance (AUC 0.675) compared to baseline CVAI-FI (AUC 0.641), standalone CVAI (AUC 0.616), and baseline FI (AUC 0.609), with significant improvements in both discrimination (IDI) and reclassification (NRI) beyond comprehensive clinical models. The associations remained remarkably consistent across diverse subgroups defined by demographic, lifestyle, and clinical characteristics. Significant effect modifications were detected for education level and hypertension status, with more pronounced associations among better-educated participants and normotensive individuals. Our findings highlight the clinical applicability of CVAI-FI—especially when evaluated over time—for identifying vulnerable individuals requiring targeted preventive measures within CKM syndrome management frameworks.

This study provides the first evidence that CVAI-FI serves as a powerful independent predictor of incident stroke in early-to-intermediate CKM syndrome, consistent with prior longitudinal data emphasizing metabolic impairment and functional vulnerability in cerebrovascular pathogenesis. Prior studies have demonstrated independent associations between elevated CVAI and heightened stroke risk among middle-aged and older Chinese adults (13,14). Similarly, studies integrating lipid-related metabolic markers with the FI have demonstrated enhanced predictive capacity for cardiovascular events, including stroke and heart disease (32). Furthermore, composite indices such as the TyG-FI and the TyG-derived visceral adiposity index have demonstrated enhanced prognostic capacity for CVD and cardiometabolic multimorbidity relative to single-domain measures (29,31). The observed consistency across these investigations reinforces the validity of integrating metabolic and frailty domains for cerebrovascular risk stratification. Despite this overall concordance, important distinctions exist between the current study and prior studies. Most previous investigations focused either on insulin resistance-related indices or visceral adiposity markers alone, or examined frailty as an independent modifier, without explicitly integrating these domains into a unified composite exposure or situating the analysis within the emerging CKM syndrome framework (14,15,32). Differences in study design may also partially explain variations in effect magnitude across studies, including differences in outcome definitions (composite cardiovascular events versus stroke-specific endpoints), population characteristics (general middle-aged adults versus CKM stage-stratified populations), and exposure assessment strategies (single-point baseline measurements versus longitudinal or cumulative indices). Notably, many earlier studies relied on cross-sectional designs or short follow-up durations, limiting causal inference and the ability to capture long-term risk accumulation (13).

Beyond confirming prior observations, the current study offers several substantive methodological and conceptual advances. Using a large, nationally representative prospective cohort with extended follow-up (CHARLS 2011–2020), we demonstrated a clear dose-response relationship between CVAI-FI and stroke risk using RCS modeling, providing stronger epidemiological evidence than previously reported. Moreover, by incorporating cumulative CVAI-FI exposure derived from repeated measurements across multiple survey waves, our analyses move beyond static risk assessment and explicitly capture sustained cardiometabolic-frailty burden over time, which showed superior discriminative and reclassification performance compared with baseline indices alone (cumulative CVAI-FI AUC: 0.675 versus baseline CVAI: 0.616 or FI: 0.609). This dynamic approach addresses the limitation of single-point measurements that fail to account for temporal fluctuations in metabolic and physiological status. Additionally, unlike prior studies that examined isolated metabolic or frailty constructs, our work uniquely integrates visceral adiposity and frailty within the CKM staging system, thereby aligning risk stratification with contemporary pathophysiological frameworks and clinical prevention paradigms. Furthermore, our findings demonstrated significant improvements in IDI and NRI over traditional clinical models and individual components, highlighting the incremental clinical utility of the composite index. Thus, these features highlight the unique contribution of this study—namely, demonstrating that longitudinally assessed, multidimensional cardiometabolic-frailty burden provides incremental and clinically meaningful information for early stroke risk identification in CKM syndrome populations that is not available from existing single-domain or cross-sectional approaches. The observed effect modifications warrant cautious interpretation. The stronger association among individuals with higher education may reflect a relative dilution of CVAI-FI’s predictive contribution in lower-education groups who bear a greater burden of competing stroke risk factors, or differential patterns of health reporting and healthcare access across educational strata. The more pronounced association in normotensive individuals suggests that CVAI-FI may capture metabolic-frailty-related cerebrovascular risk that is unmasked in the absence of dominant hypertension-driven risk. However, given that multiple interaction tests were conducted across 12 subgroups, the possibility that some of the observed effect modifications represent chance findings cannot be excluded, despite the relatively small *P* values for interaction (education: *P <* 0.001; hypertension: *P* = 0.002 for baseline CVAI-FI). Independent replication in other cohorts is warranted to confirm these subgroup-specific patterns.

Certain methodological considerations should be acknowledged when interpreting these data. The observational study design precludes definitive causal inference. Although we employed extensive covariate adjustment, sensitivity analyses, and E-value calculations to assess robustness to unmeasured confounding, residual confounding cannot be entirely excluded. Additionally, frailty itself may reflect underlying subclinical vascular disease burden rather than functioning solely as an independent upstream exposure, raising the possibility of bidirectional associations between frailty and cerebrovascular pathology. Stroke ascertainment relied on participant-reported physician diagnoses rather than systematic adjudication using medical records or neuroimaging, potentially introducing outcome misclassification bias. While validation studies in CHARLS have demonstrated reasonable concordance between self-reported and clinician-verified cardiovascular diagnoses (33,38), non-differential misclassification may have attenuated the observed associations. The restriction to Chinese participants limits generalizability to other ethnic groups with different body composition, adipose tissue distribution, and metabolic profiles. Given that CVAI was specifically developed for Chinese populations and ethnic variations in visceral adiposity-related risk are well documented, external validation in diverse populations is essential. Additionally, the study did not differentiate between ischemic and hemorrhagic stroke, which possess distinct pathophysiological pathways and risk profiles, thereby limiting etiology-specific risk assessment. Additionally, the CVAI-FI composite index involves multiplying two variables with inherently different measurement scales (CVAI: unbounded, range approximately –20 to 330; FI: bounded 0–1), which may cause the index to be primarily driven by CVAI. However, sensitivity analyses using alternative formulations—including a sum-based composite [z(CVAI) + z(FI)]—yielded consistent results (Supplementary Table S3), and this multiplicative approach is consistent with established precedent in published studies employing analogous metabolic-frailty composite indices (31,32,39,40). Despite these limitations, our findings provide valuable evidence supporting integrated metabolic-frailty assessment for stroke risk stratification in CKM syndrome populations. Future research should prioritize adjudicated stroke outcomes with subtype classification, validation in ethnically diverse cohorts, and intervention trials targeting combined metabolic-frailty optimization to establish whether reducing CVAI-FI translates into stroke risk reduction. Additionally, mechanistic studies exploring pathways linking visceral adiposity and frailty to cerebrovascular events could elucidate biological targets for preventive interventions and advance clinical translation into routine risk assessment strategies.

Several research directions merit prioritization to advance clinical translation of CVAI-FI. Prospective studies incorporating rigorous stroke adjudication through systematic medical record review and neuroimaging verification would strengthen causal inference, with explicit differentiation of ischemic and hemorrhagic subtypes to enable etiology-specific risk prediction (41). Multi-ethnic validation studies are essential to assess cross-population generalizability, particularly given that CVAI was developed specifically for Chinese populations. Randomized controlled trials evaluating whether targeted CVAI-FI reduction through combined lifestyle modification and metabolic management translates into measurable stroke risk reduction would provide definitive evidence for therapeutic benefit. Beyond traditional risk factor modification, mechanistic investigations employing multi-omics approaches could elucidate biological pathways linking visceral adiposity-frailty interactions to cerebrovascular pathogenesis, particularly the roles of adipokine dysregulation, chronic inflammation, and accelerated biological aging (42). Given that cumulative CVAI-FI demonstrated incremental predictive value over baseline assessment, future investigations should explore dynamic risk prediction models incorporating time-varying CVAI-FI trajectories with machine learning algorithms capable of capturing non-linear temporal patterns and identifying critical windows where preventive interventions yield maximal benefit (43). From a clinical implementation perspective, integration of CVAI-FI screening into existing CKM syndrome staging algorithms warrants systematic evaluation. Development and validation of simplified CVAI-FI calculators or digital health applications enabling point-of-care risk estimation could facilitate widespread adoption in primary care settings, particularly in resource-limited environments. Extension of the CVAI-FI framework to predict other CKM-related outcomes—including heart failure, chronic kidney disease progression, and all-cause mortality—would comprehensively characterize its prognostic value across the full spectrum of cardiometabolic disease. These research directions converge toward a paradigm shift from single-domain risk assessment to multidimensional vulnerability profiling, potentially transforming stroke prevention from reactive treatment of established risk factors to proactive optimization of metabolic resilience throughout the life course.

## Conclusion

Among participants with early-to-intermediate CKM syndrome (stages 0–3), higher CVAI-FI is independently and nonlinearly associated with elevated stroke probability. Cumulative CVAI-FI assessment exhibits superior discriminative performance compared to baseline CVAI-FI and individual components, with significant improvements in both discrimination (IDI) and reclassification (NRI) beyond comprehensive clinical models. By combining metabolic impairment with functional vulnerability assessment, CVAI-FI delivers improved risk stratification versus isolated risk factors, maintaining consistent associations across heterogeneous demographic and clinical subpopulations. These observations validate CVAI-FI’s clinical applicability, particularly when measured cumulatively, for identifying at-risk individuals warranting targeted prevention efforts in CKM syndrome contexts.

## Additional File

The additional file for this article can be found as follows:

10.5334/gh.1597.s1 Supplementary File. Supplementary Tables S1 to S16 and Figures S1 to S9.

## Data Availability

The data underlying this article are available from the China Health and Retirement Longitudinal Study (CHARLS) public database at https://charls.pku.edu.cn and can be accessed upon registration and approval.
