# Supplementary material for: Association Between the Chinese Visceral Adiposity Index-Frailty Index (CVAI-FI) and the Incidence of New-Onset Stroke in Individuals with Cardiovascular-Kidney-Metabolic Syndrome Stage 0–3: Evidence From a National Population-Based Study
Source: Glob Heart. 2026 Oct 22;21(1):75. doi: 10.5334/gh.1597 (PMC13693587; doi:10.5334/gh.1597)
Supplement: Supplementary file 1 [file gh-21-1-1597-s1.pdf]

Supplementary materials

Table S1. The 32 items used to construct the frailty index (FI)

No	Description of the items		Cut-off value
1	Self-reported physician diagnosed hypertension		Yes = 1, No = 0
2	Self-reported physician diagnosed diabetes		Yes = 1, No = 0
3	Self-reported physician diagnosed heart disease		Yes = 1, No = 0
4	Self-reported physician diagnosed stroke		Yes = 1, No = 0
5	Self-reported physician diagnosed cancer		Yes = 1, No = 0
6	Self-reported physician diagnosed arthritis		Yes = 1, No = 0
7	Self-reported physician diagnosed chronic lung disease		Yes = 1, No = 0
8	Self-reported physician diagnosed asthma		Yes = 1, No = 0
9	Self-reported physician diagnosed any emotional, nervous, or psychiatric problems		Yes = 1, No = 0
10	Self-reported physician diagnosed memory-related disease		Yes = 1, No = 0
11	Self-reported vision problems	Self-reported eyesight (while using lenses if appropriate)	Yes = 1, No = 0
12	Self-reported hearing problems	Self-reported hearing (while using hearing aid if appropriate)	Yes = 1, No = 0
13	Self-reported general health status		Poor or fair = 1, excellent, very good, or good = 0
14	Difficulty with dressing		Yes = 1, No = 0
15	Difficulty with bathing or showering		Yes = 1, No = 0
16	Difficulty with eating		Yes = 1, No = 0
17	Difficulty with getting in and out of bed		Yes = 1, No = 0
18	Difficulty with using the toilet		Yes = 1, No = 0
19	Difficulty with managing money		Yes = 1, No = 0
20	Difficulty with taking medications		Yes = 1, No = 0
21	Difficulty with shopping for groceries		Yes = 1, No = 0
22	Difficulty with preparing meals		Yes = 1, No = 0
23	Difficulty with doing housework		Yes = 1, No = 0
24	Mobility: difficulty with walking 100 yards		Yes = 1, No = 0
25	Mobility: difficulty with getting up from a chair after sitting for long periods		Yes = 1, No = 0
26	Mobility: difficulty with climbing several flights of stairs without resting		Yes = 1, No = 0
27	Mobility: difficulty with lifting or carrying weights over 10 pounds/jins		Yes = 1, No = 0
28	Mobility: difficulty with picking up a coin from the table		Yes = 1, No = 0
29	Mobility: difficulty with stooping, kneeling, or crouching		Yes = 1, No = 0
30	Mobility: difficulty with reaching arms above shoulder level		Yes = 1, No = 0
31	Depression: CESD-10 questionnaire		CESD-10 >10 =1, ≤10 =0

32	Cognition: (memory test score + orientation test score) / 14	Continuous, ranging from 0 to 1

To maximize analytical sample size while maintaining data quality, we employed the following strategies for handling missing FI data: participants with more than 10% missing values across the 32 FI variables were excluded from the analysis; for participants with ≤10% missing data, remaining missing values were imputed using multiple imputation by chained equations, generating five complete datasets which were subsequently pooled for final FI calculation. Heart disease includes angina pectoris, coronary artery disease, congestive heart failure, and other cardiac conditions. Memory-related disorders include Alzheimer's disease, dementia, organic brain syndrome, and other significant cognitive impairments. Depressive symptoms were assessed using the Center for Epidemiologic Studies Depression Scale (CESD-10) in CHARLS, with scores ranging from 0 to 30, where higher scores reflect greater symptom severity. Memory function was quantified as the mean number of words correctly recalled across immediate and delayed word recall tests (score range: 0-10). Cognitive orientation was evaluated through four items assessing awareness of the current day of the week, month, date, and year, with one point assigned for each incorrect response (score range: 0-4).

Table S2. Formal interaction analysis comparing model specifications incorporating the Chinese Visceral Adiposity Index (CVAI) and Frailty Index (FI) for stroke risk prediction in individuals with Cardiovascular-Kidney-Metabolic Syndrome Stage 0-3.

Model specification	OR (95% CI) per SD	AUC (95% CI)	AIC	LRT P value
Model A: CVAI only	1.29 (1.16–1.44)***	0.691 (0.668–0.714)	3148.08	—
Model B: FI only	1.28 (1.15–1.41)***	0.699 (0.676–0.722)	3148.36	—
Model C: CVAI + FI (main effects)	CVAI: 1.29 (1.16–1.44)***<br>FI: 1.28 (1.15–1.41)***	0.706 (0.683–0.728)	3129.34	Ref.
Model D: CVAI + FI + CVAI×FI (interaction)	CVAI: 1.29 (1.16–1.44)***<br>FI: 1.28 (1.16–1.41)***<br>CVAI×FI: 0.96 (0.88–1.04)	0.706 (0.683–0.728)	3130.32	0.312
Model E: CVAI-FI composite	1.29 (1.18–1.41)***	0.702 (0.679–0.724)	3139.30	—
LRT: Model C vs Model D (interaction test)				0.312
LRT: Model A vs Model C (FI added)				<0.001
LRT: Model B vs Model C (CVAI added)				<0.001

Abbreviations: OR, odds ratio; CI, confidence interval; AUC, area under the receiver operating characteristic curve; AIC, Akaike Information Criterion; LRT, likelihood ratio test; SD, standard deviation; CVAI, Chinese Visceral Adiposity Index; FI, Frailty Index; CVAI-FI, Chinese Visceral Adiposity Index–Frailty Index composite. All models were adjusted for age, sex, education level, smoking status, marital status, drinking status, fasting blood glucose, glycated hemoglobin, total cholesterol, low-density lipoprotein cholesterol, estimated glomerular filtration rate, systolic blood pressure, diabetes, cancer, chronic lung disease, liver disease, kidney disease, lipid-lowering medication use, health insurance status, and residential area (hukou). CVAI and FI were standardized to per-SD units before entry into regression models. The CVAI×FI interaction term was calculated as the product of standardized CVAI and standardized FI. The CVAI-FI composite index was derived as the product of raw CVAI and FI values (CVAI × FI) and subsequently standardized. P values for individual coefficients were derived from Wald tests. LRT P values compare the specified nested models. *** $P < 0.001$ for individual OR estimates.

Table S3. Sensitivity analysis comparing alternative formulations of the Chinese Visceral Adiposity Index-Frailty Index (CVAI-FI) composite for stroke risk prediction in individuals with Cardiovascular-Kidney-Metabolic Syndrome Stage 0–3.

Composite formulation	Formula	OR (95% CI) per SD	AUC (95% CI)	AIC	Correlation with original CVAI-FI
Original CVAI-FI (current approach)	CVAI × FI	1.293 (1.182–1.411)***	0.702 (0.679–0.724)	3139.30	1.00 (reference)
Standardized product	z(CVAI) × z(FI)	1.051 (0.962–1.146) P = 0.264	0.682 (0.659–0.706)	3168.03	0.43
Sum-based composite	z(CVAI) + z(FI)	1.418 (1.277–1.573)***	0.706 (0.683–0.729)	3127.40	0.91
CVAI alone	CVAI (per SD)	1.293 (1.162–1.440)***	0.691 (0.668–0.714)	3148.08	0.89
FI alone	FI (per SD)	1.279 (1.155–1.413)***	0.699 (0.676–0.722)	3148.36	0.40

Abbreviations: OR, odds ratio; CI, confidence interval; AUC, area under the receiver operating characteristic curve; AIC, Akaike Information Criterion; SD, standard deviation; CVAI, Chinese Visceral Adiposity Index; FI, Frailty Index; z(·), z-score standardization (mean = 0, SD = 1).

All models were adjusted for age, sex, education level, smoking status, marital status, drinking status, fasting blood glucose, glycated hemoglobin, total cholesterol, low-density lipoprotein cholesterol, estimated glomerular filtration rate, systolic blood pressure, diastolic blood pressure, hypertension, diabetes, cancer, chronic lung disease, liver disease, kidney disease, lipid-lowering medication use, health insurance status, and residential area (hukou). The original CVAI-FI is calculated as the raw product of CVAI and FI. The standardized product replaces each component with its z-score before multiplication. The sum-based composite adds the z-scores of CVAI and FI. CVAI alone and FI alone are presented for comparison. Correlation coefficients represent Pearson r between each alternative formulation and the original CVAI-FI. *** $P < 0.001$ for individual OR estimates. P values for the standardized product were not significant ($P = 0.264$), likely reflecting mathematical instability when multiplying two z-scores near zero.

Table S4. Methods for staging CKM stages 0-4

CKM stages	Threshold for CKM conditions
Stages 0	All criteria are met:<br>① BMI< 23 kg/m^2<br>② Waist circumference <80/90 cm in female/male<br>③ Fasting blood glucose < 100 mg/dL and HbA1c < 5.7% and without self-reported diagnosis of diabetes, use of insulin, or oral hypoglycemic agents.<br>④ SBP <130 mm Hg and DBP <80 mm Hg without self-reported diagnosis of hypertension or use of antihypertensive medications.<br>⑤ HDL-C ≥50/40 mg/dL in female/male<br>⑥ TG < 150 mg/dL<br>⑦ eGFR ≥ 60 ml/min/1.73m^2 and without self-reported diagnosis of CKD<br>⑧ No Subclinical CVD and clinical CVD
Stages 1	Any of the three criteria is met:<br>① Overweight/obesity<br>② Abdominal obesity<br>③ Prediabetes<br>All criteria are met:<br>(1) SBP<130 mmHg and DBP<80 mmHg without self-reported diagnosis of hypertension or use of antihypertensive medications.<br>(2) HDL-C <50/40 mg/dL in female/male<br>(3) TG < 150 mg/dL<br>(4) eGFR ≥ 60 ml/min/1.73m^2 and without self-reported diagnosis of CKD<br>(5) No Subclinical CVD and clinical CVD
Stages 2	Any of the five criteria is met:<br>① Hypertriglyceridemia<br>② Hypertension<br>③ Diabetes<br>④ metabolic syndrome<br>⑤ eGFR: 30-60 ml/min/1.73m^2 and/or with self-reported diagnosis of CKD<br>All criteria are met:<br>① No Subclinical CVD and clinical CVD
Stages 3	Any of the two criteria is met:<br>① eGFR<30 ml/min/1.73m^2<br>② Subclinical CVD<br>Any of the eight criteria is met:<br>① Overweight/obesity<br>② Abdominal obesity<br>③ Prediabetes<br>④ Hypertriglyceridemia<br>⑤ Hypertension<br>⑥ diabetes<br>⑦ metabolic syndrome<br>⑧ eGFR: 30-60 ml/min/1.73m^2 and/or with self-reported diagnosis of CKD

	The criterion is met:<br>① No clinical CVD
Stages 4	Clinical CVD: (self-reported diagnosed cardiovascular disease, including heart failure, coronary heart disease, angina, heart attack, and stroke) in individuals

Abbreviation: CVD: cardiovascular diseases; CKM: Cardiovascular–kidney–metabolic Syndrome; SBP: systolic blood pressure; DBP: diastolic blood pressure; TC: total cholesterol; HDL: high-density lipoprotein cholesterol; LDL: low-density lipoprotein cholesterol; eGFR: estimated glomerular filtration ratio; TG: triglyceride; BMI: body mass index; CKD: chronic kidney disease.

Table S5. Specific definitions of various diseases

Overweight/obesity	BMI ≥23 kg/m^2
Abdominal obesity	Waist circumference≥80/90 cm in female/male
Prediabetes	Fasting blood glucose ≥ 100-124 mg/dL or HbA1c ≥ 5.7%-6.4% and without self-reported diagnosis of diabetes, use of insulin, or oral hypoglycemic agents
Diabetes	Fasting blood glucose ≥ 125 mg/dL or HbA1c ≥ 6.5% or self-reported diagnosis of diabetes, use of insulin, or oral hypoglycemic agents
Prediabetes	Fasting blood glucose ≥ 100-124 mg/dL or HbA1c ≥ 5.7%-6.4% and without self-reported diagnosis of diabetes, use of insulin, or oral hypoglycemic agents
Hypertension	SBP ≥130 mm Hg or DBP ≥80 mm Hg or self-reported diagnosis of hypertension or use of antihypertensive medications
Chronic kidney disease	Any of the two criteria is met:<br>① Self-reported history of chronic kidney disease<br>② eGFR < 60 ml/min/1.73m^2
Hypertriglyceridemia	Triglycerides ≥ 135 mg/dL
Metabolic syndrome	Meet any three or more of the five<br>① Waist circumference ≥ 80/90 cm in female/male.<br>②HDL cholesterol < 50/40 mg/dL in female/male.<br>③ Triglycerides ≥135 mg/dL.<br>④ Elevated blood pressure (SBP ≥130 mm Hg or DBP ≥80 mm Hg and/or use of antihypertensive medications)<br>⑤ Fasting blood glucose ≥100 mg/dL
Clinical CVD	Any of the two criteria is met:<br>① Participants were asked "Have you been told by a doctor that you have been diagnosed with a heart disease, including angina, heart attack, congestive heart failure, and other heart problems?"<br>②"Have you been told by a doctor that you have been diagnosed with a stroke?"
Subclinical CVD	Any of the two criteria is met:<br>① Very high-risk CKD in KDIGO classification: eGFR < 30 ml/min/1.73m^2.<br>② Framingham risk score≥21.5/21.6 in female/male

Abbreviation: CVD: cardiovascular diseases; CKD: chronic kidney disease; HbA1c: Glycated Hemoglobin; SBP: systolic blood pressure; DBP: diastolic blood pressure; TC: total cholesterol; HDL: high-density lipoprotein cholesterol; eGFR: estimated glomerular filtration ratio; BMI: body mass index; KDIGO: Kidney Disease: Improving Global Outcomes.

Table S6 Baseline characteristics comparison between included participants vs excluded participants for baseline CVAI-FI

Characteristics	Overall (n=17705)[1]	Included (n=6772)[1]	Excluded (n=10933)[1]	*P* Value[2]
Age, years	59.05 ± 10.16	58.95 ± 9.26	59.11 ± 10.68	0.3
Sex, n (%)				0.009
Missing	12 (0.1%)	0 (0.0%)	12 (0.1%)	
Female	9,222 (52.1%)	3,574 (52.8%)	5,648 (51.7%)	
Male	8,471 (47.8%)	3,198 (47.2%)	5,273 (48.2%)	
Education level				<0.001
Missing	52 (0.3%)	2 (0.0%)	50 (0.5%)	
Higher	858 (4.8%)	200 (3.0%)	658 (6.0%)	
Primary school	11,755 (66.4%)	4,716 (69.6%)	7,039 (64.4%)	
Secondary school	5,040 (28.5%)	1,854 (27.4%)	3,186 (29.1%)	
Height, cm	157.67 ± 11.42	157.97 ± 8.58	157.37 ± 13.63	0.002
Weight, kg	58.76 ± 11.73	58.58 ± 11.11	58.94 ± 12.31	0.076
BMI, kg/m²	445.39 ± 10,043.09	23.42 ± 3.75	862.25 ± 14,148.17	<0.001
Waist circumference, cm	84.28 ± 12.62	84.97 ± 9.83	83.61 ± 14.80	<0.001
SBP, mmHg	129.52 ± 21.48	128.56 ± 20.89	130.44 ± 22.00	<0.001
DBP, mmHg	75.45 ± 12.23	74.99 ± 12.00	75.90 ± 12.43	<0.001
FBG, mmol/L	110.30 ± 37.33	108.93 ± 33.93	112.21 ± 41.54	<0.001
HbA1c, %	5.26 ± 0.82	5.26 ± 0.81	5.26 ± 0.85	0.8
TC, mmol/L	192.97 ± 38.89	194.10 ± 38.17	191.40 ± 39.83	<0.001
HDL-C, mmol/L	50.84 ± 15.33	51.71 ± 15.20	49.64 ± 15.43	<0.001
LDL-C, mmol/L	115.99 ± 34.91	117.17 ± 34.69	114.37 ± 35.15	<0.001
TG, mmol/L	134.91 ± 110.26	129.22 ± 103.54	142.80 ± 118.51	<0.001
eGFR, ml/min/1.73m²	92.23 ± 15.25	92.75 ± 14.47	91.48 ± 16.25	<0.001
CVAI	842.72 ± 26,003.76	94.68 ± 39.28	2,540.91 ± 46,985.62	0.004
Frailty Index	0.16 ± 0.14	0.15 ± 0.11	0.17 ± 0.15	<0.001
Frailty, n (%)				<0.001
No	14,082 (80.0%)	5,723 (84.5%)	8,359 (77.2%)	
Yes	3,512 (20.0%)	1,049 (15.5%)	2,463 (22.8%)	
Hypertension, n (%)				<0.001
No	8,239 (55.9%)	4,257 (62.9%)	3,982 (50.0%)	
Yes	6,494 (44.1%)	2,506 (37.1%)	3,988 (50.0%)	
Diabetes, n (%)				<0.001
No	15,194 (86.5%)	5,757 (85.0%)	9,437 (87.5%)	
Yes	2,366 (13.5%)	1,015 (15.0%)	1,351 (12.5%)	
Cancer, n (%)				0.030
No	17,304 (99.0%)	6,706 (99.2%)	10,598 (98.8%)	
Yes	180 (1.0%)	55 (0.8%)	125 (1.2%)	
Chronic lung disease, n (%)				<0.001
No	15,713 (89.8%)	6,157 (91.0%)	9,556 (89.1%)	
Yes	1,781 (10.2%)	609 (9.0%)	1,172 (10.9%)	
Liver disease, n (%)				0.3
No	16,760 (96.1%)	6,508 (96.3%)	10,252 (96.0%)	

Yes	676 (3.9%)	249 (3.7%)	427 (4.0%)	
Kidney disease, n (%)				0.051
No	16,344 (93.7%)	6,373 (94.1%)	9,971 (93.4%)	
Yes	1,106 (6.3%)	398 (5.9%)	708 (6.6%)	
Lipid-lowering drugs, n (%)				<0.001
No	16,792 (94.8%)	6,525 (96.4%)	10,267 (93.9%)	
Yes	913 (5.2%)	247 (3.6%)	666 (6.1%)	
Diabetes drugs, n (%)				0.2
No	876 (87.7%)	294 (89.9%)	582 (86.6%)	
Yes	123 (12.3%)	33 (10.1%)	90 (13.4%)	
CKM, n (%)				<0.001
NA	9,097 (51.4%)	0 (0.0%)	9,097 (83.2%)	
0	316 (1.8%)	280 (4.1%)	36 (0.3%)	
1	829 (4.7%)	749 (11.1%)	80 (0.7%)	
2	1,708 (9.6%)	1,573 (23.2%)	135 (1.2%)	
3	4,400 (24.9%)	4,170 (61.6%)	230 (2.1%)	
4	1,355 (7.7%)	0 (0.0%)	1,355 (12.4%)	
Smoking status, n (%)				0.5
No	10,626 (60.5%)	4,122 (60.9%)	6,504 (60.3%)	
Yes	6,931 (39.5%)	2,649 (39.1%)	4,282 (39.7%)	
Married, n (%)				<0.001
No	2,258 (12.8%)	776 (11.5%)	1,482 (13.6%)	
Yes	15,417 (87.2%)	5,996 (88.5%)	9,421 (86.4%)	
Drinking status, n (%)				0.8
No	10,716 (81.1%)	4,069 (81.2%)	6,647 (81.0%)	
Yes	2,501 (18.9%)	942 (18.8%)	1,559 (19.0%)	
Heart disease, n (%)				<0.001
No	15,478 (88.8%)	6,772 (100.0%)	8,706 (81.4%)	
Yes	1,987 (11.2%)	0 (0.0%)	1,987 (18.6%)	
Residence (urban), n (%)				<0.001
No	8,377 (47.3%)	2,892 (42.7%)	5,485 (50.2%)	
Yes	9,328 (52.7%)	3,880 (57.3%)	5,448 (49.8%)	
Health insurance, n (%)				<0.001
No	1,180 (6.7%)	371 (5.5%)	809 (7.4%)	
Yes	16,525 (93.3%)	6,401 (94.5%)	10,124 (92.6%)	

[1]Mean ± SD; n (%)

[2]Welch Two Sample t-test; Pearson's Chi-squared test

Table S7 Baseline characteristics comparison between included participants vs excluded participants for cumulative CVAI-FI

Characteristics	Overall (n=17705)[1]	Included (n=4170)[1]	Excluded (n=13535)[1]	*P* Value[2]
Age, years	59.05 ± 10.16	58.32 ± 8.54	59.28 ± 10.60	<0.001
Unknown	23	0	23	
Sex, n (%)				<0.001
Missing	12 (0.1%)	1 (0.0%)	12 (0.1%)	
Female	9,222 (52.1%)	2,269 (54.4%)	6,953 (51.4%)	
Male	8,471 (47.8%)	1,901 (45.6%)	6,570 (48.5%)	
Education level				<0.001
Missing	52 (0.3%)	0 (0.0%)	51 (0.4%)	
Higher	858 (4.8%)	108 (2.6%)	750 (5.5%)	
Primary school	11,755 (66.4%)	2,884 (69.2%)	8,871 (65.5%)	
Secondary school	5,040 (28.5%)	1,177 (28.2%)	3,863 (28.5%)	
Smoking status, n (%)				0.006
No	10,626 (60.5%)	2,600 (62.4%)	8,026 (60.0%)	
Yes	6,931 (39.5%)	1,570 (37.6%)	5,361 (40.0%)	
Unknown	148	0	148	
Married, n (%)				<0.001
No	2,258 (12.8%)	414 (9.9%)	1,844 (13.7%)	
Yes	15,417 (87.2%)	3,756 (90.1%)	11,661 (86.3%)	
Unknown	30	0	30	
Drinking status, n (%)				0.4
No	10,716 (81.1%)	2,549 (81.6%)	8,167 (80.9%)	
Yes	2,501 (18.9%)	573 (18.4%)	1,928 (19.1%)	
Unknown	4,488	1,048	3,440	
Residence (urban), n (%)				<0.001
No	8,377 (47.3%)	1,705 (40.9%)	6,672 (49.3%)	
Yes	9,328 (52.7%)	2,465 (59.1%)	6,863 (50.7%)	
Health insurance, n (%)				<0.001
No	1,180 (6.7%)	202 (4.8%)	978 (7.2%)	
Yes	16,525 (93.3%)	3,968 (95.2%)	12,557 (92.8%)	
Height, cm	157.67 ± 11.42	158.12 ± 8.41	157.47 ± 12.50	<0.001
Unknown	4,010	0	4,010	
Weight, kg	58.76 ± 11.73	59.08 ± 10.91	58.62 ± 12.07	0.029
Unknown	3,981	0	3,981	
BMI, kg/m²	445.39 ± 10,043.09	23.59 ± 3.79	631.38 ± 12,051.16	<0.001
Unknown	4,078	0	4,078	
Waist circumference, cm	84.28 ± 12.62	85.32 ± 9.78	83.82 ± 13.65	<0.001
Unknown	3,936	0	3,936	
SBP, mmHg	129.52 ± 21.48	127.70 ± 20.20	130.31 ± 21.97	<0.001
Unknown	3,998	23	3,975	
DBP, mmHg	75.45 ± 12.23	74.80 ± 11.79	75.73 ± 12.41	<0.001

Unknown	3,998	23	3,975	
FBG, mmol/L	110.30 ± 37.33	108.53 ± 33.07	111.29 ± 39.48	<0.001
Unknown	6,069	0	6,069	
HbA1c, %	5.26 ± 0.82	5.26 ± 0.79	5.26 ± 0.84	0.9
Unknown	5,999	0	5,999	
TC, mmol/L	192.97 ± 38.89	193.91 ± 37.80	192.44 ± 39.48	0.048
Unknown	6,050	0	6,050	
HDL-C, mmol/L	50.84 ± 15.33	51.20 ± 15.09	50.65 ± 15.45	0.060
Unknown	6,042	0	6,042	
LDL-C, mmol/L	115.99 ± 34.91	117.12 ± 34.41	115.37 ± 35.17	0.009
Unknown	6,063	5	6,058	
TG, mmol/L	134.91 ± 110.26	130.86 ± 104.13	137.16 ± 113.48	0.002
Unknown	6,049	0	6,049	
eGFR, ml/min/1.73m²	92.23 ± 15.25	93.72 ± 13.79	91.39 ± 15.95	<0.001
Unknown	6,153	0	6,153	
Hypertension, n (%)				<0.001
No	8,239 (55.9%)	2,661 (63.9%)	5,578 (52.8%)	
Yes	6,494 (44.1%)	1,502 (36.1%)	4,992 (47.2%)	
Unknown	2,972	7	2,965	
Diabetes, n (%)				0.004
No	15,194 (86.5%)	3,552 (85.2%)	11,642 (86.9%)	
Yes	2,366 (13.5%)	618 (14.8%)	1,748 (13.1%)	
Unknown	145	0	145	
Heart disease, n (%)				<0.001
No	15,372 (88.0%)	4,170 (100.0%)	11,271 (84.8%)	
Yes	2,093 (12.0%)	0	2,024 (15.2%)	
Unknown	240	0	240	
Cancer, n (%)				0.3
No	17,304 (99.0%)	4,125 (99.1%)	13,179 (98.9%)	
Yes	180 (1.0%)	36 (0.9%)	144 (1.1%)	
Unknown	221	9	212	
Chronic lung disease, n (%)				0.002
No	15,713 (89.8%)	3,795 (91.1%)	11,918 (89.4%)	
Yes	1,781 (10.2%)	371 (8.9%)	1,410 (10.6%)	
Unknown	211	4	207	
Liver disease, n (%)				0.029
No	16,760 (96.1%)	4,022 (96.7%)	12,738 (95.9%)	
Yes	676 (3.9%)	137 (3.3%)	539 (4.1%)	
Unknown	269	11	258	
Kidney disease, n (%)				0.002
No	16,344 (93.7%)	3,948 (94.7%)	12,396 (93.3%)	
Yes	1,106 (6.3%)	221 (5.3%)	885 (6.7%)	
Unknown	255	1	254	
Lipid-lowering drugs, n (%)				<0.001

No	16,792 (94.8%)	3,999 (95.9%)	12,793 (94.5%)	
Yes	913 (5.2%)	171 (4.1%)	742 (5.5%)	
Diabetes drugs, n (%)				0.2
No	876 (87.7%)	191 (90.5%)	685 (86.9%)	
Yes	123 (12.3%)	20 (9.5%)	103 (13.1%)	
Unknown	16,706	3,959	12,747	
CVAI (2011)	842.72 ± 26,003.76	95.39 ± 38.75	1,400.71 ± 34,357.42	0.005
Unknown	7,950	0	7,950	
CKM, n (%)				<0.001
Missing	9,097 (51.4%)	0 (0.0%)	9,097 (67.2%)	
0	316 (1.8%)	172 (4.1%)	144 (1.1%)	
1	829 (4.7%)	509 (12.2%)	320 (2.4%)	
2	1,708 (9.6%)	1,029 (24.7%)	679 (5.0%)	
3	4,400 (24.9%)	2,460 (59.0%)	1,940 (14.3%)	
4	1,355 (7.7%)	0 (0.0%)	1,355 (10.0%)	
Frailty Index (32-item, 2011)	0.16 ± 0.14	0.14 ± 0.11	0.17 ± 0.14	<0.001
Unknown	111	0	111	
Frailty (2011), n (%)				<0.001
No	14,082 (80.0%)	3,579 (85.8%)	10,503 (78.2%)	
Yes	3,512 (20.0%)	591 (14.2%)	2,921 (21.8%)	
Unknown	111	0	111	
CVAI (2015)	103.95 ± 39.21	100.27 ± 35.29	107.21 ± 33.28	<0.001
Unknown	13,535	0	13,535	
Frailty Index (32-item, 2015)	0.20 ± 0.15	0.18 ± 0.13	0.21 ± 0.15	<0.001
Unknown	3,176	0	3,176	
Frailty (2015), n (%)				<0.001
No	10,473 (72.1%)	3,231 (77.5%)	7,242 (69.9%)	
Yes	4,056 (27.9%)	939 (22.5%)	3,117 (30.1%)	
Unknown	3,176	0	3,176	

[1]Mean ± SD; n (%)

[2]Welch Two Sample t-test; Pearson's Chi-squared test

Table S8 Association between the Chinese visceral adiposity index (CVAI)-frailty index (FI) and stroke incidence in individuals with Cardiovascular-Kidney-Metabolic Syndrome Stage 0-3 after excluding participants with missing data on covariates

Characteristic	Model 1 OR (95%CI), *P*	Model 2 OR (95%CI), *P*	Model 3 OR (95%CI), *P*
CVAI-FI Per unit increase	1.030 (1.024, 1.036), <0.0001	1.024 (1.016, 1.031), <0.0001	1.021 (1.014, 1.028), <0.0001
CVAI-FI Per SD increase	1.520 (1.394, 1.656), <0.0001	1.393 (1.261, 1.536), <0.0001	1.345 (1.212, 1.490), <0.0001
CVAI-FI quartile			
Q1	1 (Reference)	1 (Reference)	1 (Reference)
Q2	1.565 (1.052, 2.354)*	1.468 (0.982, 2.215)	1.413 (0.944, 2.138)
Q3	1.990 (1.361, 2.951)**	1.771 (1.197, 2.655)**	1.661 (1.117, 2.503)*
Q4	3.753 (2.651, 5.426)**	2.834 (1.935, 4.224)**	2.458 (1.656, 3.711)**
P for trend	<0.0001	<0.0001	<0.0001

Model 1: unadjusted for any covariates; Model 2: adjusted for gender, age, residence, education level, marital status, smoking status, drinking status, insurance status, and systolic blood pressure (SBP); Model 3: adjusted for gender, age, residence, education level, marital status, smoking status, and drinking status, insurance status, systolic blood pressure (SBP), Glycated hemoglobin (HBA1c), fasting blood glucose (FBG), diabetes, cancer, liver cancer, kidney disease, chronic lung disease, total cholesterol (TC), low-density lipoprotein cholesterol (LDL-C), estimated glomerular filtration rate (eGFR), and lipid-lowering drugs. Note: *P <0.05, **P<0.01

Table S9 Association between the cumulative Chinese visceral adiposity index-frailty index (CVAI-FI) and stroke incidence in individuals with Cardiovascular-Kidney-Metabolic Syndrome Stage 0–3 after excluding all missing data

Characteristic	Model 1 OR (95%CI), *P*	Model 2 OR (95%CI), *P*	Model 3 OR (95%CI), *P*
Cumulative CVAI-FI Per unit increase	1.012 (1.010, 1.015), <0.0001	1.011 (1.008, 1.014), <0.0001	1.011 (1.007, 1.014), <0.0001
Cumulative CVAI-FI Per SD increase	1.637 (1.485, 1.802), <0.0001	1.541 (1.359, 1.745), <0.0001	1.542 (1.349, 1.761), <0.0001
Cumulative CVAI-FI quartile			
Q1	1 (Reference)	1 (Reference)	1 (Reference)
Q2	2.138 (1.318, 3.562)**	1.288 (0.721, 2.345)	1.263 (0.704, 2.307)
Q3	2.503 (1.563, 4.131)**	1.650 (0.948, 2.954)	1.578 (0.898, 2.851)
Q4	5.840 (3.812, 9.329)**	3.744 (2.230, 6.551)**	3.432 (2.002, 6.112)**
P for trend	<0.0001	<0.0001	<0.0001

Model 1: unadjusted for any covariates; Model 2: adjusted for gender, age, residence, education level, marital status, smoking status, drinking status, insurance status, systolic blood pressure (SBP); Model 3: adjusted for gender, age, residence, education level, marital status, smoking status, drinking status, insurance status, systolic blood pressure (SBP), Glycated hemoglobin (HBA1c), fasting blood glucose (FBG), cancer, liver cancer, kidney disease, chronic lung disease, total cholesterol (TC), low-density lipoprotein cholesterol (LDL-C), estimated glomerular filtration rate (eGFR), and lipid-lowering drugs. Note: **P* <0.05, ***P*<0.01

Table S10 Association between the Chinese visceral adiposity index (CVAI)-frailty index and stroke incidence in individuals with Cardiovascular-Kidney-Metabolic Syndrome Stage 0-3 without prevalent stroke at wave 2

Characteristic	Model 1 OR (95%CI), *P*	Model 2 OR (95%CI), *P*	Model 3 OR (95%CI), *P*
CVAI-FI Per unit increase	1.026 (1.020, 1.032), <0.0001	1.021 (1.014, 1.027), <0.0001	1.019 (1.012, 1.026), <0.0001
CVAI-FI Per SD increase	1.429 (1.320, 1.545), <0.0001	1.320 (1.204, 1.444), <0.0001	1.290 (1.171, 1.417), <0.0001
CVAI-FI quartile			
Q1	1 (Reference)	1 (Reference)	1 (Reference)
Q2	1.762 (1.227, 2.559)**	1.644 (1.141, 2.394)**	1.598 (1.107, 2.330)*
Q3	2.347 (1.663, 3.360)**	2.107 (1.479, 3.045)**	2.009 (1.404, 2.912)**
Q4	3.623 (2.617, 5.107)**	2.837 (1.991, 4.107)**	2.580 (1.789, 3.774)**
P for trend	<0.0001	<0.0001	<0.0001

Model 1: unadjusted for any covariates; Model 2: adjusted for gender, age, residence, education level, marital status, smoking status, and drinking status, insurance status, systolic blood pressure (SBP); Model 3: adjusted for gender, age, residence, education level, marital status, smoking status, and drinking status, insurance status, systolic blood pressure (SBP), Glycated hemoglobin (HBA1c), fasting blood glucose (FBG), diabetes, cancer, liver cancer, kidney disease, chronic lung disease, total cholesterol (TC), low-density lipoprotein cholesterol (LDL-C), estimated glomerular filtration rate (eGFR), lipid-lowering drugs. Note: *P <0.05, **P<0.01

Table S11 Association between the Chinese visceral adiposity index-frailty index (CVAI-FI) and stroke incidence in individuals with Cardiovascular-Kidney-Metabolic Syndrome Stage 0-3 excluding drinking status as a covariate

Characteristic	Model 1 OR (95%CI), *P*	Model 2 OR (95%CI), *P*	Model 3 OR (95%CI), *P*
CVAI-FI Per unit increase	1.030 (1.024, 1.036), <0.0001	1.024 (1.017, 1.031), <0.0001	1.021 (1.014, 1.029), <0.0001
CVAI-FI Per SD increase	1.520 (1.394, 1.656), <0.0001	1.399 (1.267, 1.542), <0.0001	1.349 (1.216, 1.494), <0.0001
CVAI-FI quartile			
Q1	1 (Reference)	1 (Reference)	1 (Reference)
Q2	1.565 (1.052, 2.354)*	1.483 (0.993, 2.237)	1.424 (0.951, 2.154)
Q3	1.990 (1.361, 2.951)**	1.798 (1.217, 2.693)**	1.680 (1.131, 2.529)*
Q4	3.753 (2.651, 5.426)**	2.904 (1.989, 4.316)**	2.504 (1.691, 3.770)**
P for trend	<0.0001	<0.0001	<0.0001

Model 1: unadjusted for any covariates; Model 2: adjusted for gender, age, residence, education level, marital status, smoking status, insurance status, systolic blood pressure (SBP); Model 3: adjusted for gender, age, residence, education level, marital status, smoking status, insurance status, systolic blood pressure (SBP), Glycated hemoglobin (HBA1c), fasting blood glucose (FBG), diabetes, cancer, liver cancer, kidney disease, chronic lung disease, total cholesterol (TC), low-density lipoprotein cholesterol (LDL-C), estimated glomerular filtration rate (eGFR), lipid-lowering drugs. Note: *P <0.05, **P<0.01

Table S12 Association between the cumulative Chinese visceral adiposity index-frailty index (CVAI-FI) and stroke incidence in individuals with Cardiovascular-Kidney-Metabolic Syndrome Stage 0-3 excluding drinking status as a covariate

Characteristic	Model 1 OR (95%CI), *P*	Model 2 OR (95%CI), *P*	Model 3 OR (95%CI), *P*
Cumulative CVAI-FI Per unit increase	1.012 (1.010, 1.015), <0.0001	1.011 (1.008, 1.014), <0.0001	1.011 (1.008, 1.014), <0.0001
Cumulative CVAI-FI Per SD increase	1.637 (1.485, 1.802), <0.0001	1.550 (1.387, 1.729), <0.0001	1.541 (1.369, 1.732), <0.0001
Cumulative CVAI-FI quartile			
Q1	1 (Reference)	1 (Reference)	1 (Reference)
Q2	2.138 (1.318, 3.562)**	2.053 (1.260, 3.435)**	1.989 (1.218, 3.333)**
Q3	2.503 (1.563, 4.131)**	2.268 (1.396, 3.791)**	2.162 (1.323, 3.634)**
Q4	5.840 (3.812, 9.329)**	4.879 (3.070, 8.036)**	4.520 (2.797, 7.551)**
P for trend	<0.0001	<0.0001	<0.0001

Model 1: unadjusted for any covariates; Model 2: adjusted for gender, age, residence, education level, marital status, smoking status, insurance status, systolic blood pressure (SBP); Model 3: adjusted for gender, age, residence, education level, marital status, smoking status, insurance status, systolic blood pressure (SBP), Glycated hemoglobin (HBA1c), fasting blood glucose (FBG), cancer, liver cancer, kidney disease, chronic lung disease, total cholesterol (TC), low-density lipoprotein cholesterol (LDL-C), estimated glomerular filtration rate (eGFR), and lipid-lowering drugs. Note: *P <0.05, **P<0.01

Table S13 Association between the Chinese visceral adiposity index (CVAI)-frailty index and stroke incidence in individuals with Cardiovascular-Kidney-Metabolic Syndrome Stage 0-3 excluding prevalent hypertension at baseline

Characteristic	Model 1 OR (95%CI), *P*	Model 2 OR (95%CI), *P*	Model 3 OR (95%CI), *P*
CVAI-FI Per unit increase	1.033 (1.024, 1.043), <0.0001	1.030 (1.019, 1.040), <0.0001	1.030 (1.019, 1.041), <0.0001
CVAI-FI Per SD increase	1.457 (1.312, 1.612), <0.0001	1.398 (1.241, 1.569), <0.0001	1.407 (1.242, 1.589), <0.0001
CVAI-FI quartile			
Q1	1 (Reference)	1 (Reference)	1 (Reference)
Q2	1.523 (0.948, 2.466)	1.471 (0.912, 2.394)	1.457 (0.902, 2.375)
Q3	2.154 (1.374, 3.429)**	2.060 (1.293, 3.329)**	2.049 (1.280, 3.325)**
Q4	4.142 (2.693, 6.501)**	3.814 (2.374, 6.234)**	3.842 (2.366, 6.337)**
P for trend	<0.0001	<0.0001	<0.0001

Model 1: unadjusted for any covariates; Model 2: adjusted for gender, age, residence, education level, marital status, smoking status, insurance status, systolic blood pressure (SBP); Model 3: adjusted for gender, age, residence, education level, marital status, smoking status, insurance status, systolic blood pressure (SBP), Glycated hemoglobin (HBA1c), fasting blood glucose (FBG), diabetes, cancer, liver cancer, kidney disease, chronic lung disease, total cholesterol (TC), low-density lipoprotein cholesterol (LDL-C), estimated glomerular filtration rate (eGFR), lipid-lowering drugs. Note: **P* <0.05, ***P*<0.01

Table S14 Association between the cumulative Chinese visceral adiposity index-frailty index (CVAI-FI) and stroke incidence in individuals with Cardiovascular-Kidney-Metabolic Syndrome Stage 0-3 excluding prevalent hypertension at baseline

Characteristic	Model 1 OR (95%CI), *P*	Model 2 OR (95%CI), *P*	Model 3 OR (95%CI), *P*
Cumulative CVAI-FI Per unit increase	1.011 (1.007, 1.015), <0.0001	1.010 (1.005, 1.015), <0.0001	1.011 (1.006, 1.016), <0.0001
Cumulative CVAI-FI Per SD increase	1.478 (1.283, 1.693), <0.0001	1.410 (1.197, 1.652), <0.0001	1.456 (1.228, 1.718), <0.0001
Cumulative CVAI-FI quartile			
Q1	1 (Reference)	1 (Reference)	1 (Reference)
Q2	2.037 (1.030, 4.248)*	2.015 (1.011, 4.230)	2.038 (1.020, 4.286)*
Q3	2.751 (1.442, 5.604)**	2.621 (1.350, 5.416)**	2.671 (1.369, 5.543)**
Q4	3.955 (2.141, 7.890)**	3.446 (1.772, 7.163)**	3.700 (1.883, 7.753)**
P for trend	<0.0001	<0.0001	<0.0001

Model 1: unadjusted for any covariates; Model 2: adjusted for gender, age, residence, education level, marital status, smoking status, drinking status, insurance status, systolic blood pressure (SBP); Model 3: adjusted for gender, age, residence, education level, marital status, smoking status, drinking status, insurance status, systolic blood pressure (SBP), Glycated hemoglobin (HBA1c), fasting blood glucose (FBG), diabetes, cancer, liver cancer, kidney disease, chronic lung disease, total cholesterol (TC), low-density lipoprotein cholesterol (LDL-C), estimated glomerular filtration rate (eGFR), and lipid-lowering drugs. Note: *P <0.05, **P<0.01

Table S15 Association between the Chinese visceral adiposity index (CVAI)-frailty index and stroke incidence in individuals with Cardiovascular-Kidney-Metabolic Syndrome Stage 0–3 after excluding prevalent diabetes at baseline

Characteristic	Model 1 OR (95%CI), P	Model 2 OR (95%CI), P	Model 3 OR (95%CI), P
Per unit increase	1.028 (1.021, 1.034), <0.0001	1.021 (1.013, 1.029), <0.0001	1.019 (1.011, 1.027), <0.0001
Per SD increase	1.422 (1.307, 1.542), <0.0001	1.310 (1.188, 1.440), <0.0001	1.276 (1.154, 1.408), <0.0001
CVAI-FI quartile			
Q1	1 (Reference)	1 (Reference)	1 (Reference)
Q2	1.933 (1.331, 2.844)**	1.817 (1.246, 2.684)**	1.745 (1.195, 2.582)**
Q3	2.517 (1.757, 3.664)**	2.308 (1.592, 3.396)**	2.148 (1.475, 3.173)**
Q4	3.798 (2.688, 5.469)**	3.074 (2.100, 4.568)**	2.778 (1.880, 4.165)**
P for trend	<0.0001	<0.0001	<0.0001

Model 1: unadjusted for any covariates; Model 2: adjusted for gender, age, residence, education level, marital status, smoking status, drinking status, insurance status, systolic blood pressure (SBP); Model 3: adjusted for gender, age, residence, education level, marital status, smoking status, drinking status, insurance status, systolic blood pressure (SBP), Glycated hemoglobin (HBA1c), fasting blood glucose (FBG), cancer, liver cancer, kidney disease, chronic lung disease, total cholesterol (TC), low-density lipoprotein cholesterol (LDL-C), estimated glomerular filtration rate (eGFR), and lipid-lowering drugs. Note: **P* <0.05, ***P*<0.01

Table S16 Association between the cumulative Chinese visceral adiposity index-frailty index (CVAI-FI) and stroke incidence in individuals with Cardiovascular-Kidney-Metabolic Syndrome Stage 0-3 excluding prevalent diabetes at baseline

Characteristic	Model 1 OR (95%CI), *P*	Model 2 OR (95%CI), *P*	Model 3 OR (95%CI), *P*
Cumulative CVAI-FI Per unit increase	1.012 (1.009, 1.014), <0.0001	1.010 (1.007, 1.013), <0.0001	1.010 (1.007, 1.014), <0.0001
Cumulative CVAI-FI Per SD increase	1.560 (1.397, 1.737), <0.0001	1.478 (1.303, 1.671), <0.0001	1.477 (1.296, 1.679), <0.0001
Cumulative CVAI-FI quartile			
Q1	1 (Reference)	1 (Reference)	1 (Reference)
Q2	2.671 (1.543, 4.838)**	2.576 (1.481, 4.687)**	2.548 (1.461, 4.646)**
Q3	2.799 (1.623, 5.056)**	2.565 (1.464, 4.698)**	2.520 (1.429, 4.639)**
Q4	5.707 (3.457, 9.997)**	4.830 (2.807, 8.751)**	4.784 (2.747, 8.754)**
P for trend	<0.0001	<0.0001	<0.0001

Model 1: unadjusted for any covariates; Model 2: adjusted for gender, age, residence, education level, marital status, smoking status, drinking status, insurance status, systolic blood pressure (SBP); Model 3: adjusted for gender, age, residence, education level, marital status, smoking status, drinking status, insurance status, systolic blood pressure (SBP), Glycated hemoglobin (HBA1c), fasting blood glucose (FBG), cancer, liver cancer, kidney disease, chronic lung disease, total cholesterol (TC), low-density lipoprotein cholesterol (LDL-C), estimated glomerular filtration rate (eGFR), and lipid-lowering drugs. Note: *P <0.05, **P<0.01

Figure S1 Distribution of missing values across study variables

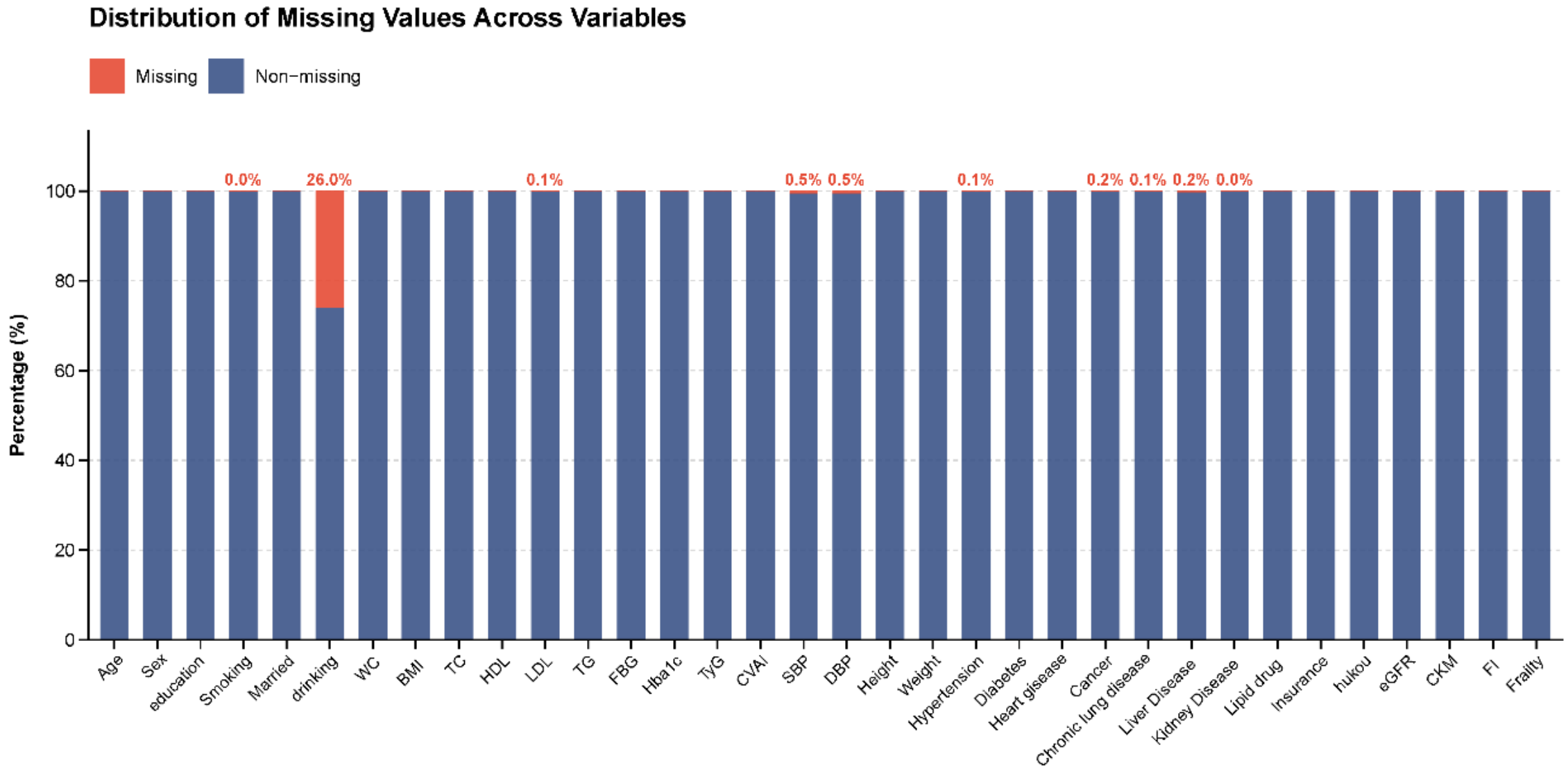

Bar chart displaying the percentage of missing data for each variable included in the baseline analysis cohort (N = 6,772). The x-axis shows all study variables, and the y-axis represents the percentage of missing values (0-100%). Each bar is color-coded with red indicating missing data and blue indicating non-missing data. Percentages are labeled above bars with missing rates ≥0.1%. Drinking status exhibited the highest proportion of missing data at 26.0%, while all other variables demonstrated missing rates below 1%: HbA1c (0.5%), CVAI (0.5%), eGFR (0.2%), hypertension (0.2%), heart disease (0.2%), liver disease (0.2%), waist circumference (0.1%), HDL-C (0.1%), LDL-C (0.1%), FBG (0.1%), diabetes (0.1%), kidney disease (0.1%), height (0.1%), with the remaining variables showing 0.0% missing. Given that alcohol consumption is an established stroke risk factor, we applied k-nearest neighbors (KNN) imputation for drinking status to retain the full analytical sample, while maintaining high data completeness for all other variables. Abbreviations: BMI, body mass index; WC, waist circumference; TC, total cholesterol; HDL-C, high-density lipoprotein cholesterol; LDL-C, low-density lipoprotein cholesterol; TG, triglycerides; FBG, fasting blood glucose; HbA1c, glycated hemoglobin; TyG, triglyceride-glucose index; CVAI, Chinese visceral adiposity index; SBP, systolic blood pressure; DBP, diastolic blood pressure; eGFR, estimated glomerular filtration rate; CKM, cardiovascular-kidney-metabolic syndrome; FI, frailty index.

Figure S2 Kaplan–Meier survival curves for cumulative stroke risk by the quartiles of Chinese visceral adiposity index-frailty index (CVAI-FI).

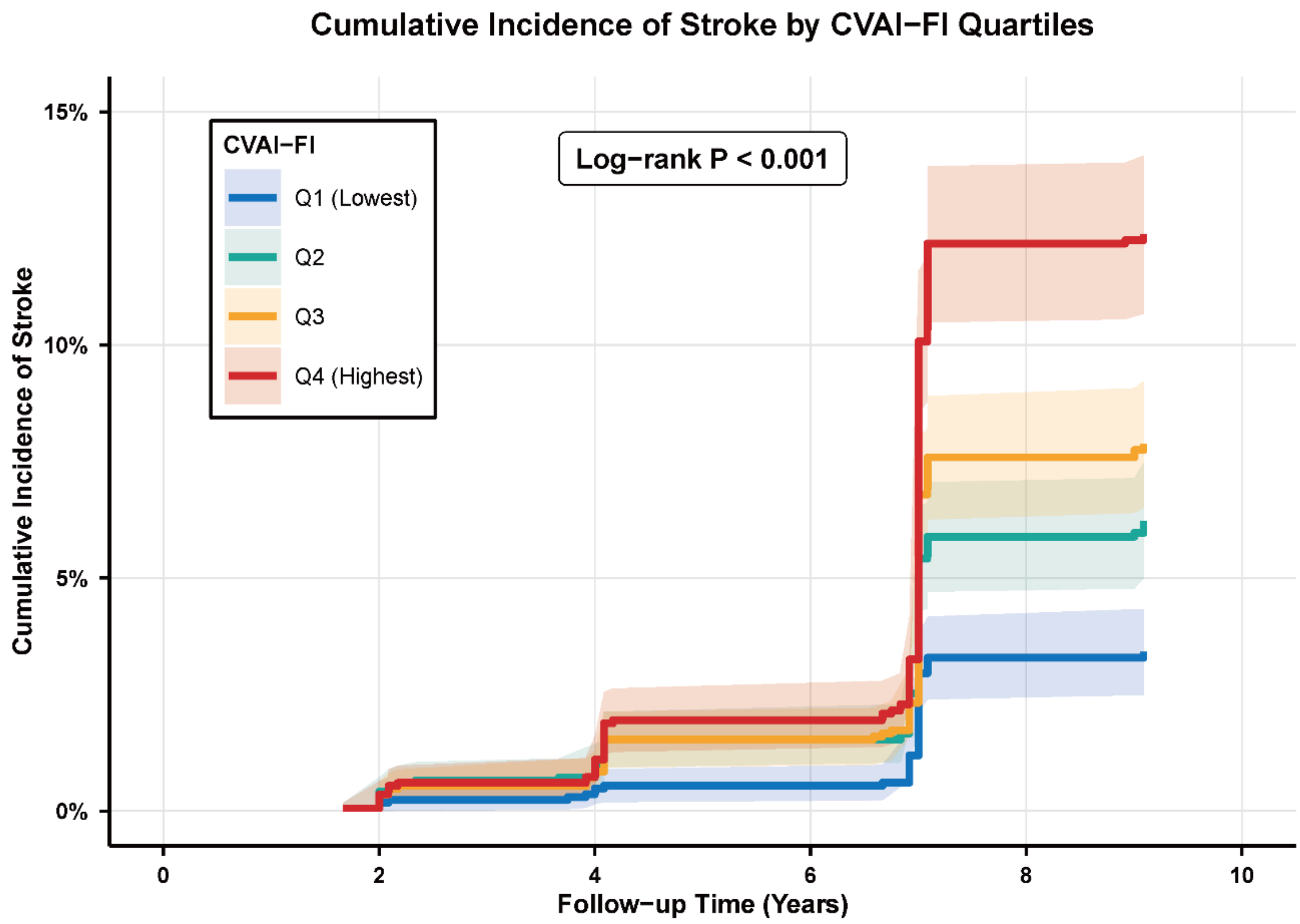

Kaplan–Meier survival curves depicting cumulative stroke-free survival probability over 9-year follow-up (2011–2020) stratified by quartiles of baseline Chinese visceral adiposity index-frailty index (CVAI-FI). Participants were categorized into four groups based on baseline CVAI-FI distribution: Q1 (lowest quartile, reference), Q2 (second quartile), Q3 (third quartile), and Q4 (highest quartile). The y-axis represents the cumulative probability of remaining stroke-free, and the x-axis represents time from baseline enrollment (years). Each colored curve corresponds to a quartile group, with shaded regions indicating 95% confidence intervals.

Figure S3 Sensitivity analysis using Cox proportional hazards regression: restricted cubic spline analysis of the relationship between baseline CVAI-FI and stroke incidence among individuals with CKM syndrome stages 0–3

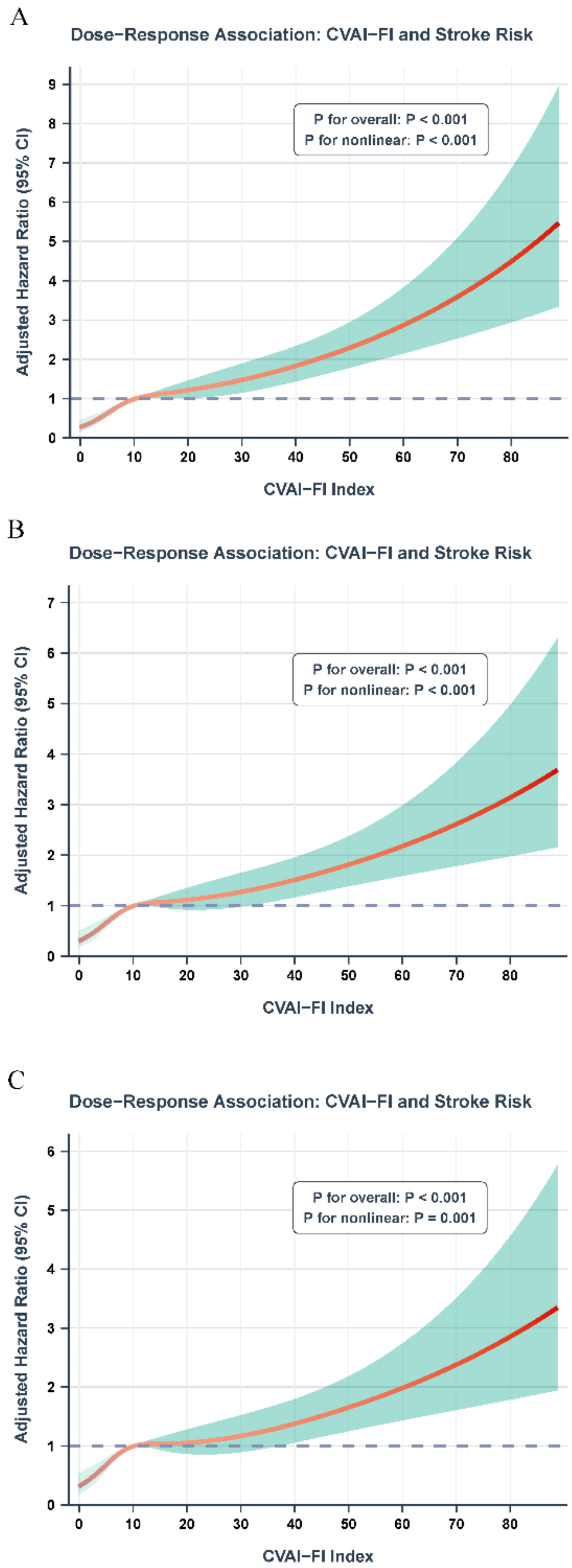

Restricted cubic spline (RCS) analysis depicting the dose-response association between the baseline Chinese visceral adiposity index-frailty index (CVAI-FI) and incident stroke risk among individuals with cardiovascular-kidney-metabolic (CKM) syndrome stages 0–3. (A) Model 1: crude model without covariate adjustment. (B) Model 2: adjusted for demographic and lifestyle factors including gender, age, residence, education level, marital status, smoking status, drinking status, insurance status, systolic blood pressure (SBP). (C) Model 3: fully adjusted model including all covariates from Model 2 plus metabolic and clinical parameters: glycated hemoglobin (HbA1c), fasting blood glucose (FBG), diabetes, cancer, liver disease, kidney disease, chronic lung disease, total cholesterol (TC), low-density lipoprotein cholesterol (LDL-C), estimated glomerular filtration rate (eGFR), and lipid-lowering medication use. All models show significant overall associations ($P < 0.001$) with evidence of nonlinearity in Models 1-3 ($P < 0.01$).

Figure S4 Sensitivity analysis using Cox proportional hazards regression: restricted cubic spline analysis of the relationship between cumulative CVAI-FI and stroke incidence among individuals with CKM syndrome stages 0–3

A

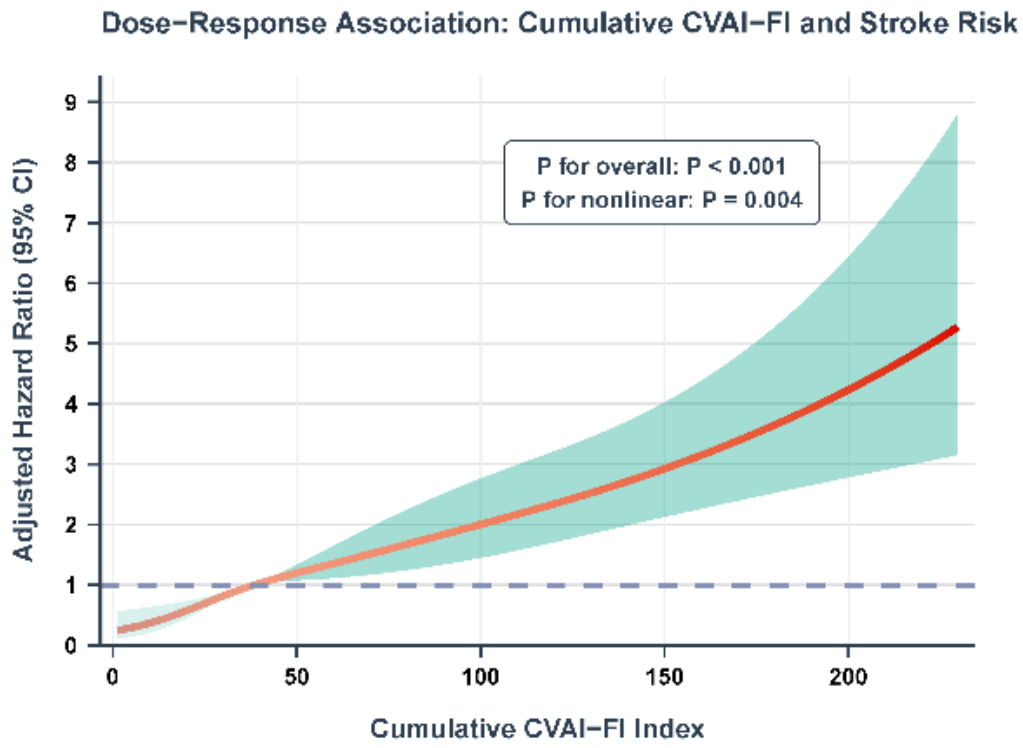

B

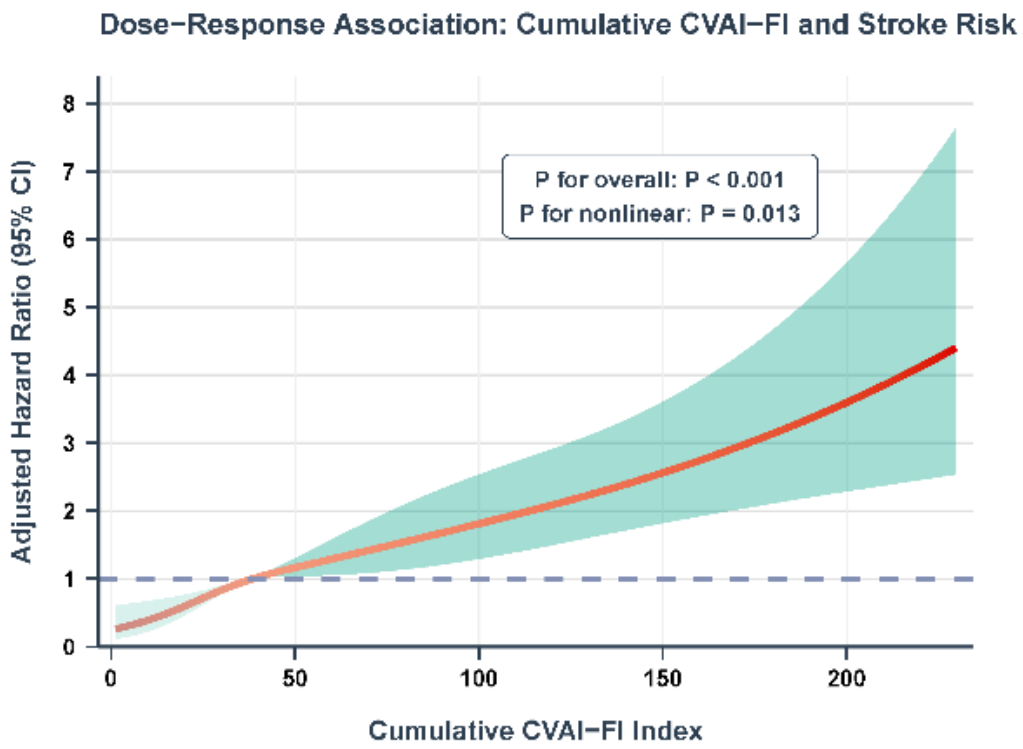

C

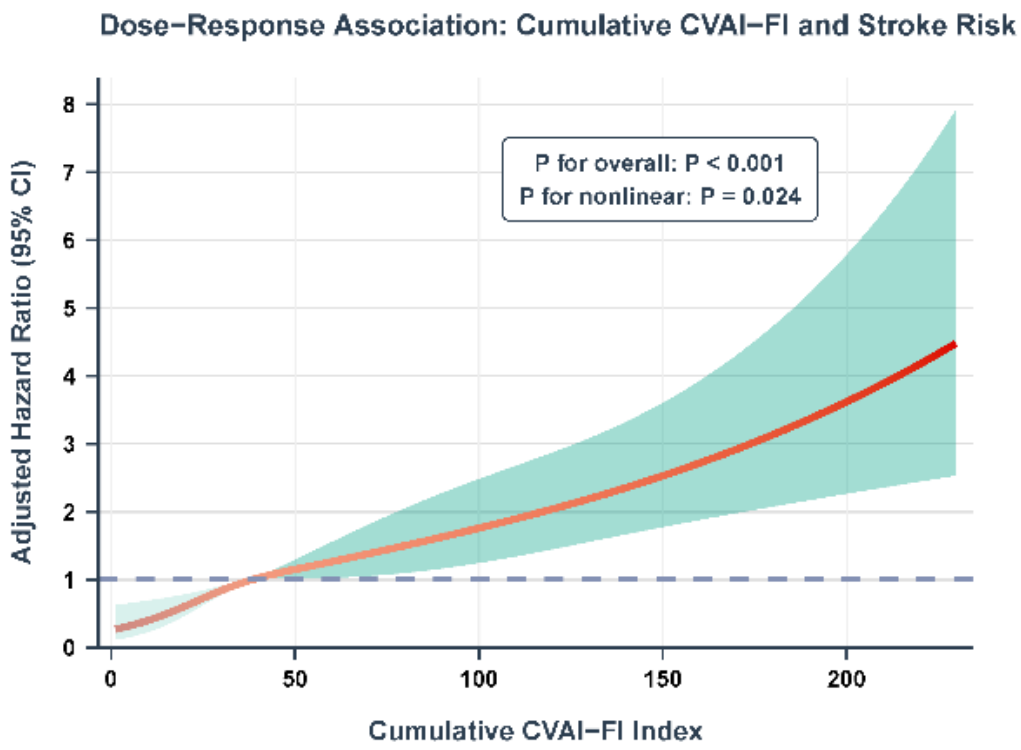

Restricted cubic spline (RCS) analysis depicting the dose-response association between the cumulative Chinese visceral adiposity index-frailty index (CVAI-FI) and incident stroke risk among individuals with cardiovascular-kidney-metabolic (CKM) syndrome stages 0-3. (A) Model 1: crude model without covariate adjustment. (B) Model 2: adjusted for demographic and lifestyle factors including gender, age, residence, education level, marital status, smoking status, drinking status, insurance status, systolic blood pressure (SBP). (C) Model 3: fully adjusted model including all covariates from Model 2 plus metabolic and clinical parameters: glycated hemoglobin (HbA1c), fasting blood glucose (FBG), diabetes, cancer, liver disease, kidney disease, chronic lung disease, total cholesterol (TC), low-density lipoprotein cholesterol (LDL-C), estimated glomerular filtration rate (eGFR), and lipid-lowering medication use. All models show significant overall associations ($P < 0.001$) with evidence of nonlinearity in Models 1-3 ($P < 0.05$).

Figure S5 Sensitivity analysis using Cox proportional hazards regression: subgroup analysis of the association between baseline CVAI-FI and stroke incidence in CKM 0-3

Subgroup	Category	N	Stroke	HR 95CI	P value	P interaction
**Age**	<60	3835	199	1.392 (1.210, 1.601)	<0.0001	0.153
	>=60	2935	248	1.245 (1.124, 1.379)	<0.0001	
**Sex**	Male	3198	229	1.332 (1.182, 1.501)	<0.0001	0.687
	Female	3572	218	1.254 (1.120, 1.404)	<0.0001	
**Education level**	Primary school	4716	324	1.209 (1.099, 1.329)	<0.0001	<0.001
	Secondary school and above	2054	123	1.707 (1.443, 2.019)	<0.0001	
**Smoking status**	No	4121	242	1.338 (1.206, 1.485)	<0.0001	0.611
	Yes	2649	205	1.235 (1.082, 1.411)	0.0018	
**Marital status**	No	775	68	1.111 (0.920, 1.342)	0.275	0.051
	Yes	5995	379	1.340 (1.224, 1.468)	<0.0001	
**Drinking status**	No	5479	336	1.296 (1.181, 1.422)	<0.0001	0.738
	Yes	1291	111	1.302 (1.087, 1.560)	0.0042	
**Diabetes**	No	5756	346	1.296 (1.176, 1.428)	<0.0001	0.575
	Yes	1014	101	1.268 (1.085, 1.482)	0.0029	
**Hypertension**	No	4264	185	1.449 (1.272, 1.651)	<0.0001	0.0081
	Yes	2506	262	1.220 (1.098, 1.355)	<0.001	
**Chronic lung disease**	No	6160	406	1.259 (1.153, 1.374)	<0.0001	0.128
	Yes	610	41	1.870 (1.424, 2.455)	<0.0001	
**Liver disease**	No	6521	428	1.290 (1.185, 1.404)	<0.0001	0.785
	Yes	249	19	1.327 (0.887, 1.986)	0.168	
**Kidney disease**	No	6372	417	1.296 (1.192, 1.410)	<0.0001	0.467
	Yes	398	30	1.289 (0.881, 1.886)	0.191	
**Residence**	Urban	3880	241	1.276 (1.140, 1.428)	<0.0001	0.684
	Rural	2890	206	1.311 (1.163, 1.477)	<0.0001	

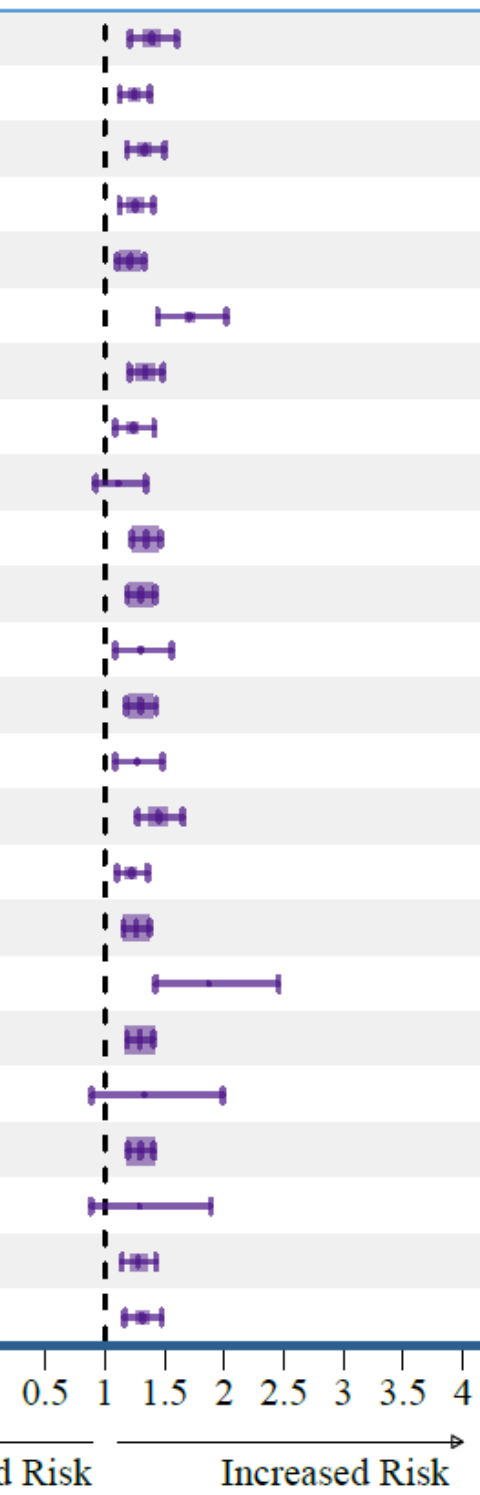

Forest plot showing subgroup-specific hazard ratios (HRs) and 95% confidence intervals (CIs) for the association between baseline CVAI-FI (per 1 SD increase) and incident stroke from Cox proportional hazards models. Subgroups defined by age, sex, education, smoking, marital status, drinking, diabetes, chronic lung disease, liver disease, kidney disease, and residence. Black squares represent HR point estimates (size proportional to precision); horizontal lines indicate 95% CIs. Vertical dashed line at HR = 1.0 represents null value. All models adjusted for demographics, lifestyle, metabolic, and clinical covariates (see Methods). *P* values test within-subgroup associations; *P* for interaction tests heterogeneity across subgroup categories. Proportional hazards assumption satisfied (Schoenfeld test, all $P > 0.05$).

Figure S6 Sensitivity analysis using Cox proportional hazards regression: subgroup analysis of the association between cumulative CVAI-FI and stroke incidence in CKM 0-3

Subgroup	Category	N	Stroke	HR 95CI	P value	P interaction
**Age**	<60	2433	118	1.748 (1.462, 2.091)	<0.0001	0.027
	>=60	1733	139	1.355 (1.187, 1.548)	<0.0001	
**Sex**	Male	1899	121	1.576 (1.334, 1.862)	<0.0001	0.489
	Female	2267	136	1.412 (1.231, 1.621)	<0.0001	
**Education level**	Primary school	2883	190	1.370 (1.213, 1.546)	<0.0001	0.0071
	Secondary school and above	1283	67	1.954 (1.577, 2.422)	<0.0001	
**Smoking status**	No	2597	155	1.507 (1.326, 1.713)	<0.0001	0.765
	Yes	1569	102	1.427 (1.187, 1.716)	<0.001	
**Marital status**	No	413	40	1.339 (0.973, 1.844)	0.073	0.05
	Yes	3753	217	1.502 (1.345, 1.678)	<0.0001	
**Drinking status**	No	3378	192	1.473 (1.308, 1.660)	<0.0001	0.993
	Yes	788	65	1.451 (1.175, 1.791)	<0.001	
**Diabetes**	No	3549	195	1.448 (1.283, 1.634)	<0.0001	0.832
	Yes	617	62	1.541 (1.238, 1.917)	<0.001	
**Hypertension**	No	2666	113	1.500 (1.258, 1.788)	<0.0001	0.61
	Yes	1500	144	1.456 (1.276, 1.661)	<0.0001	
**Chronic lung disease**	No	3794	233	1.452 (1.301, 1.622)	<0.0001	0.628
	Yes	372	24	1.898 (1.335, 2.698)	<0.001	
**Liver disease**	No	4029	249	1.473 (1.324, 1.640)	<0.0001	0.673
	Yes	137	8	2.208 (1.342, 3.634)	0.0018	
**Kidney disease**	No	3945	242	1.498 (1.346, 1.667)	<0.0001	0.128
	Yes	221	15	1.094 (0.632, 1.891)	0.749	
**Residence**	Urban	2463	143	1.516 (1.310, 1.755)	<0.0001	0.84
	Rural	1703	114	1.439 (1.239, 1.672)	<0.0001	

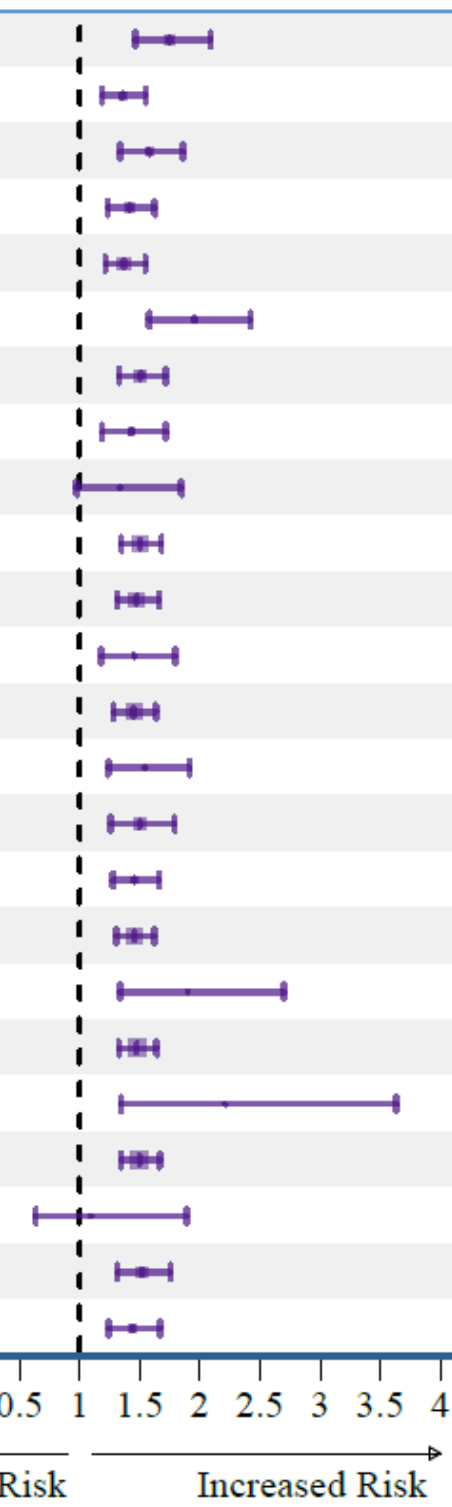

Forest plot showing subgroup-specific hazard ratios (HRs) and 95% confidence intervals (CIs) for the association between cumulative CVAI-FI (per 1 SD increase) and incident stroke from Cox proportional hazards models. Cumulative CVAI-FI was calculated as the average of CVAI-FI values measured at baseline (Wave 1, 2011) and Wave 3 (2015), representing sustained exposure over the early follow-up period. Subgroups defined by age, sex, education, smoking, marital status, drinking, diabetes, hypertension, chronic lung disease, liver disease, kidney disease, and residence. Black squares represent HR point estimates (size proportional to precision); horizontal lines indicate 95% CIs. Vertical dashed line at HR = 1.0 represents null value. All models adjusted for demographics, lifestyle, metabolic, and clinical covariates (see Methods). *P* values test within-subgroup associations; *P* for interaction tests heterogeneity across subgroup categories. Proportional hazards assumption satisfied (Schoenfeld test, all $P > 0.05$).

Figure S7 Sensitivity analysis for unmeasured confounding using E-values

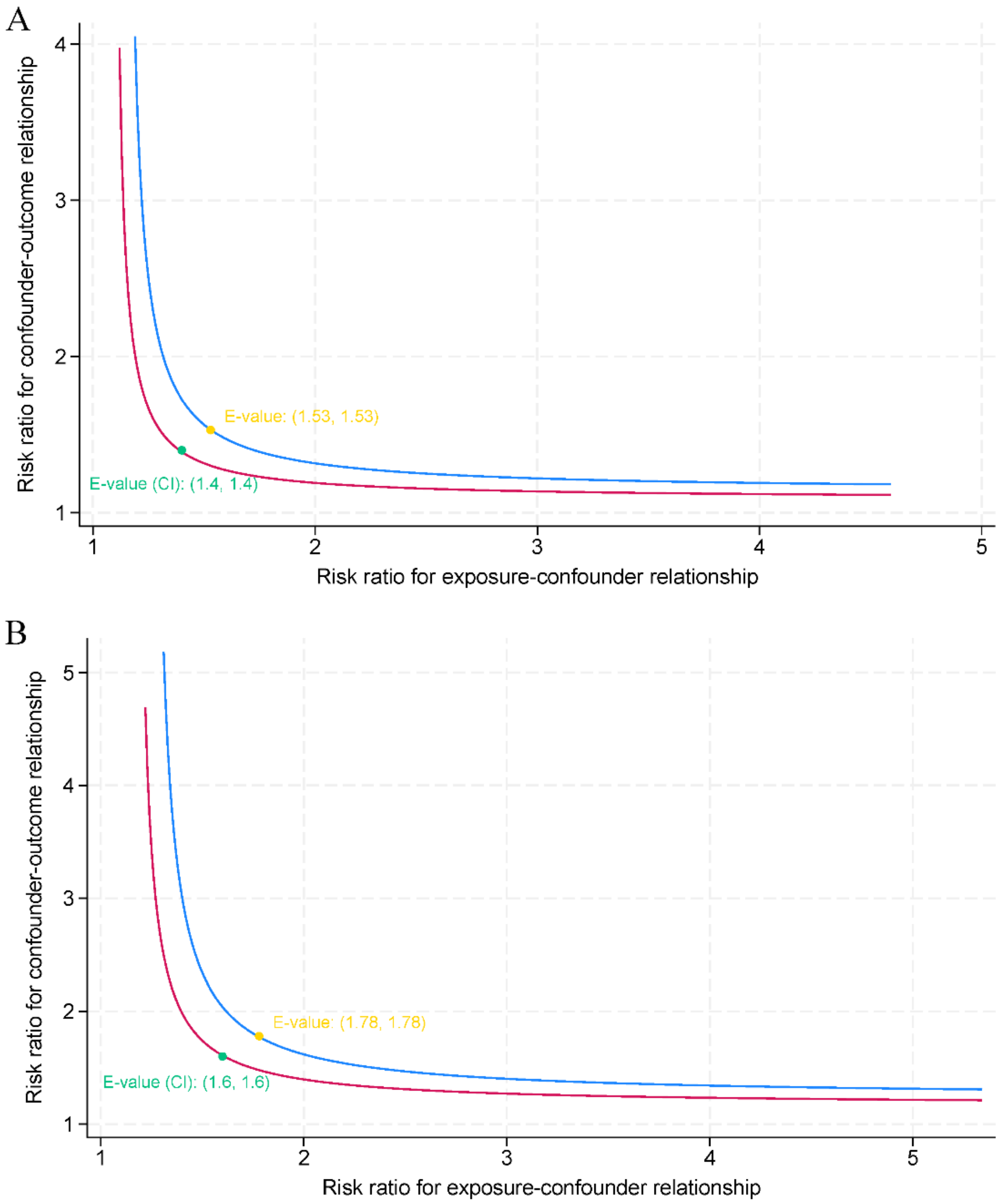

E-value analysis assessing the robustness of observed associations against potential unmeasured confounding. (A) E-value for the association between baseline CVAI-FI (per 1 standard deviation increase) and incident stroke. (B) E-value for the association between cumulative CVAI-FI (per 1 standard deviation increase) and incident stroke. The E-value represents the minimum strength of association (on the risk ratio scale) that an unmeasured confounder would need to have with both the exposure (CVAI-FI) and the outcome (stroke) to fully explain away the observed association, conditional on measured covariates. The x-axis shows the risk ratio for the confounder-outcome relationship, and the y-axis shows the risk ratio for the exposure-confounder relationship. The red curve represents the boundary beyond which unmeasured confounding could potentially nullify the observed effect. The point estimate E-value and the E-value for the confidence interval lower limit are labeled in each panel. For baseline CVAI-FI, E-value = 1.78 (95% CI lower limit: 1.60), indicating that unmeasured confounding would need to confer a risk ratio of at least 1.78-fold for both CVAI-FI and stroke to eliminate the observed association. For cumulative CVAI-FI, E-value = 1.53 (95% CI lower limit: 1.40). Higher E-values indicate greater robustness to unmeasured confounding. All analyses were based on fully adjusted models (Model 3) including demographic, lifestyle, metabolic, and clinical covariates.

Figure S8 Model performance improvement metrics for baseline CVAI-FI analysis

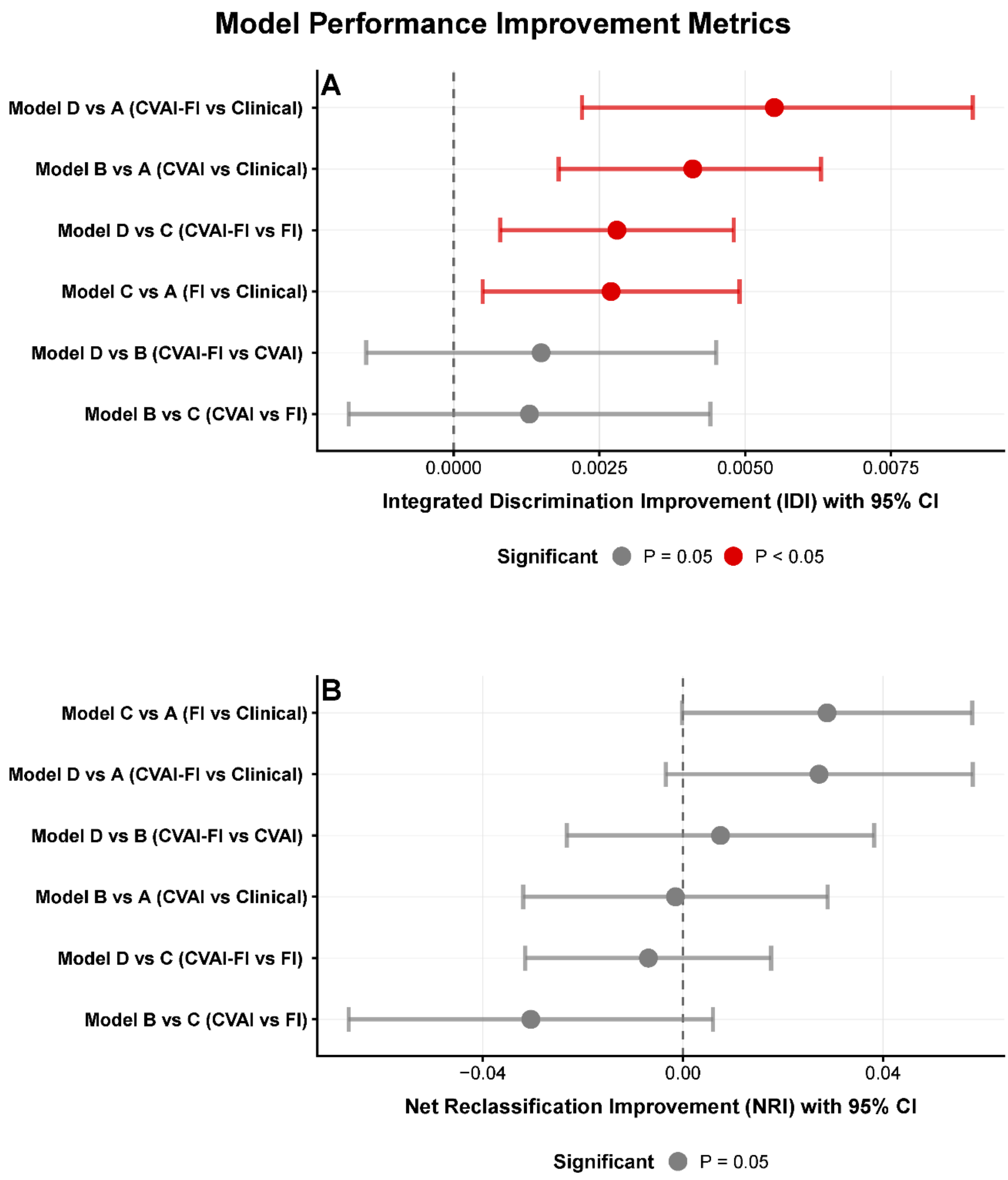

(A) Integrated Discrimination Improvement (IDI) with 95% confidence intervals comparing CVAI, FI, and CVAI-FI against Model A (clinical reference model) and their pairwise comparisons. Model A included age, sex, education, smoking status, marital status, drinking status, fasting blood glucose, HbA1c, total cholesterol, LDL-cholesterol, eGFR, systolic blood pressure, diabetes, cancer, chronic lung disease, liver disease, kidney disease, lipid-lowering medication use, insurance status, and residence. The x-axis shows IDI values. Red dots represent statistically significant improvements (P<0.05), while gray dots represent non-significant improvements (P≥0.05). Model D (CVAI-FI vs Clinical) demonstrated the largest IDI (0.005, 95% CI: 0.003–0.007, P<0.001), followed by Model B (CVAI vs Clinical): IDI=0.004 (95% CI: 0.002–0.006, P<0.001) and Model C (FI vs Clinical): IDI=0.003 (95% CI: 0.002–0.005, P<0.001). Pairwise comparisons showed: Model D vs C (CVAI-FI vs FI): IDI=0.003 (95% CI: 0.002–0.005, P<0.001); Model D vs B (CVAI-FI vs CVAI): IDI=0.002 (95% CI: 0.001–0.003,

P=0.018); Model B vs C (CVAI vs FI): IDI=0.001 (95% CI: -0.0002–0.002, P=0.065). These results indicate that CVAI-FI provides significant additive discriminative value beyond the clinical model and modest but significant incremental improvement over individual components. (B) Net Reclassification Improvement (NRI) with 95% confidence intervals. The x-axis shows NRI values; the vertical dashed line at NRI=0 represents no improvement. Most comparisons showed non-significant NRI values (gray dots). Model C (FI vs Clinical) showed the numerically largest NRI (0.032, 95% CI: -0.014–0.078, P=0.18), followed by Model D (CVAI-FI vs Clinical): NRI=0.032 (95% CI: 0.003–0.061, P=0.029); Model B (CVAI vs Clinical): NRI=0.008 (95% CI: -0.020 – 0.036, P=0.58). Pairwise comparisons showed minimal reclassification improvement: Model D vs C (CVAI-FI vs FI): NRI=-0.005 (95% CI: -0.037–0.027, P=0.76); Model D vs B (CVAI-FI vs CVAI): NRI=0.024 (95% CI: -0.004–0.052, P=0.09); Model B vs C (CVAI vs FI): NRI=-0.024 (95% CI: -0.056–0.008, P=0.14). Despite significant IDI improvements, most NRI values did not reach statistical significance, suggesting that while discrimination improved continuously, categorical risk reclassification was modest. **Abbreviations**: IDI, integrated discrimination improvement; NRI, net reclassification improvement; CVAI, Chinese visceral adiposity index; FI, frailty index; CVAI-FI, composite CVAI-FI index; Model A, clinical reference model; Model B, Model A + CVAI; Model C, Model A + FI; Model D, Model A + CVAI-FI.

Figure S9 Model performance improvement metrics for cumulative CVAI-FI analysis

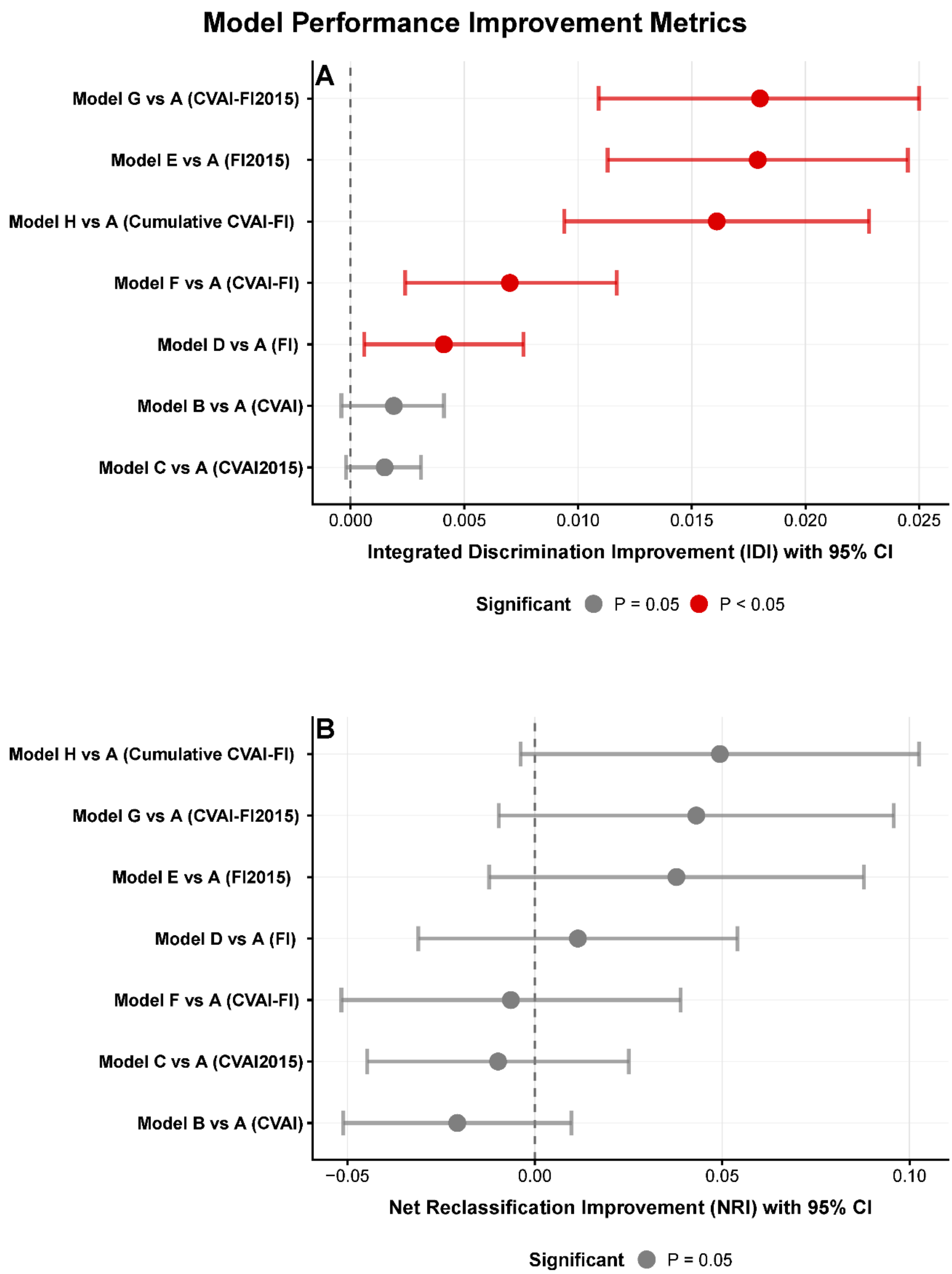

(A) Integrated Discrimination Improvement (IDI) with 95% confidence intervals comparing cumulative and time-specific CVAI-FI models against Model A (clinical reference model). Model A included age, sex, education, smoking status, marital status, drinking status, fasting blood glucose, HbA1c, total cholesterol, LDL-cholesterol, eGFR, systolic blood pressure, diabetes, cancer, chronic lung disease, liver disease, kidney disease, lipid-lowering medication use, insurance status, and

residence. The x-axis shows IDI values; positive values indicate improved discrimination. Red dots represent statistically significant improvements (*P*<0.05), while gray dots indicate non-significant improvements (*P*≥0.05). Models demonstrated the following improvements over Model A: Model G (CVAI-FI2015): IDI=0.019 (95% CI: 0.012-0.025, *P*<0.001); Model E (FI2015): IDI=0.018 (95% CI: 0.012-0.024, *P*<0.001); Model H (cumulative CVAI-FI): IDI=0.020 (95% CI: 0.013-0.026, *P*<0.001); Model F (CVAI-FI baseline): IDI=0.005 (95% CI: 0.002-0.008, *P*<0.001); Model D (FI baseline): IDI=0.004 (95% CI: 0.002-0.006, *P*<0.001). Models B (CVAI baseline) and C (CVAI2015) showed minimal non-significant improvements (IDI<0.002, *P*>0.05). (B) Net Reclassification Improvement (NRI) with 95% confidence intervals comparing the same models. The x-axis shows NRI values; the vertical dashed line at NRI=0 represents no improvement in reclassification. All models showed non-significant NRI values (gray dots, all P ≥ 0.05), suggesting that while cumulative and time-specific indices improved discrimination (IDI), they did not significantly improve categorical risk reclassification. Model H (cumulative CVAI-FI) vs Model A: NRI=0.050 (95% CI: -0.015-0.115, *P*=0.13); Model G (CVAI-FI2015) vs Model A: NRI=0.045 (95% CI: -0.020-0.110, *P*=0.18); Model E (FI2015) vs Model A: NRI=0.042 (95% CI: -0.018-0.102, *P*=0.17). **Abbreviations**: IDI, integrated discrimination improvement; NRI, net reclassification improvement; CVAI, Chinese visceral adiposity index; FI, frailty index; CVAI-FI, composite CVAI-FI index; Model A, clinical reference model; Model B, CVAI at Wave 1 (2011); Model C, CVAI at Wave 3 (2015); Model D, FI at Wave 1; Model E, FI at Wave 3; Model F, CVAI-FI at Wave 1; Model G, CVAI-FI at Wave 3; Model H, cumulative CVAI-FI (average of Wave 1 and Wave 3 measurements).
